# A systematic approach to decipher crosstalk in the p53 signaling pathway using single cell dynamics

**DOI:** 10.1371/journal.pcbi.1007901

**Published:** 2020-06-26

**Authors:** Fabian Konrath, Anna Mittermeier, Elena Cristiano, Jana Wolf, Alexander Loewer

**Affiliations:** 1 Mathematical Modelling of Cellular Processes, Max Delbrueck Center for Molecular Medicine, Berlin, Germany; 2 Systems Biology of the Stress Response, Department of Biology, Technische Universität Darmstadt, Darmstadt, Germany; 3 Signaling Dynamics in Single Cells, Berlin Institute for Medical Systems Biology, Max Delbrueck Center for Molecular Medicine, Berlin, Germany; Yale University, UNITED STATES

## Abstract

The transcription factors NF-κB and p53 are key regulators in the genotoxic stress response and are critical for tumor development. Although there is ample evidence for interactions between both networks, a comprehensive understanding of the crosstalk is lacking. Here, we developed a systematic approach to identify potential interactions between the pathways. We perturbed NF-κB signaling by inhibiting IKK2, a critical regulator of NF-κB activity, and monitored the altered response of p53 to genotoxic stress using single cell time lapse microscopy. Fitting subpopulation-specific computational p53 models to this time-resolved single cell data allowed to reproduce in a quantitative manner signaling dynamics and cellular heterogeneity for the unperturbed and perturbed conditions. The approach enabled us to untangle the integrated effects of IKK/ NF-κB perturbation on p53 dynamics and thereby derive potential interactions between both networks. Intriguingly, we find that a simultaneous perturbation of multiple processes is necessary to explain the observed changes in the p53 response. Specifically, we show interference with the activation and degradation of p53 as well as the degradation of Mdm2. Our results highlight the importance of the crosstalk and its potential implications in p53-dependent cellular functions.

## Author summary

Cells can respond to external and internal inputs by transducing information to the nucleus where transcription factors initiate corresponding cellular responses. Cellular signaling is mediated by several pathways; molecular networks that can interact with each other, which alters signal processing and modulates cellular responses. As deregulated signaling can lead to the development of tumors it is important to understand not only how signaling pathways function but also the contribution of their interaction on the signaling dynamics. Here, we analyzed the interplay of the IKK/ NF-κB and p53 pathway, which are both critical for the cellular response to DNA damage and have been implicated in tumor development. To systematically identify interaction points between both pathways, we perturbed IKK/ NF-κB signaling and tracked the changes in the response of p53 to DNA damage. Using computational methods, we show that several reactions in the p53 pathway are simultaneously affected by NF-κB signaling and that this combined action is necessary to explain altered behaviour of the p53 pathway. Hence, our results provide new insights into the interplay between the NF-κB and p53 pathway and help to gain a more comprehensive understanding of the crosstalk.

## Introduction

To adjust their physiology to varying conditions, cells need to sense a multitude of internal and external inputs, integrate the corresponding information and elicit an appropriate response. The underlying signal processing is mediated by molecular networks and their complex interactions. As interactions between networks can modulate the response of the corresponding pathways to a given stimulus [1–3], the crosstalk between pathways plays an important role in signal transduction. To gain a mechanistic understanding of signaling crosstalk, we need systematic approaches to identify potential points of interaction. Here, we developed an approach based on quantitative time-resolved measurements and mathematical modeling and chose the well characterized p53 response to DNA double strand breaks (DSBs) as a paradigm [4].

The tumor suppressor p53 is a main hub in the cellular response to numerous extrinsic and intrinsic stress factors [5]. Its crucial role as “guardian of the genome” [6] is highlighted by its frequent mutations in human cancers [7]. In the absence of stress, p53 is kept at low levels by Mdm2-mediated ubiquitination and subsequent proteasomal degradation [8]. Upon DNA damage, p53 and Mdm2 get phosphorylated by the damage-sensing kinases ATM, ATR and DNA-PK [9]. In response to DSBs, ATM also phosphorylates the checkpoint kinase Chk2, which contributes to p53 phosphorylation. Together, these post-translational modifications disrupt the p53-Mdm2 interaction and induce degradation of Mdm2 [10,11]. As a consequence, p53 accumulates in the nucleus, where it acts as a transcription factor and induces the expression of target genes mediating the cellular response to the stress [12]. These target genes include the negative regulator Mdm2 and the phosphatase Wip1, which is responsible for dephosphorylation and inactivation of ATM and its substrates including p53 [13].

The feedback loops shape the dynamics of p53 accumulation upon genotoxic stress [14–16]. Early theoretical studies and experimental evidence from cell populations suggested that p53 shows damped pulses of accumulation in damaged cells [17]. However, careful measurements of individual living cells expressing p53 fused to a fluorescent protein demonstrated that the tumor suppressor accumulates in pulses with uniform amplitude and duration as long as cells retain DSBs [18,19]. The discrepancy between results from population studies and single cell measurements could be explained by cellular heterogeneity that is caused by fluctuations in protein and mRNA levels due to intrinsic and extrinsic sources of noise and leads to asynchrony in pulse timing [20]. While intrinsic noise arises from stochasticity of biochemical processes, extrinsic noise comprises factors which have an effect on all mRNA and protein levels of an individual cell such as its volume, state or microenvironment [21]. The importance of understanding p53 dynamics is highlighted by previous observations that revealed its role in controlling cell fate upon genotoxic stress. Using genetic or pharmacological perturbations, it was shown that pulsatile p53 dynamics result in cell cycle arrest concomitant with DNA repair, whereas a sustained p53 response induces terminal cell fates like senescence or apoptosis [22,23]. Most studies of p53 dynamics combined quantitative experimentation with mathematical modeling, leading to a variety of computational approaches to represent the underlying feedback controlled molecular network [24].

Due to its central role in the stress response, the p53 network is heavily influenced by crosstalk with other signaling networks allowing cells to adjust their response to specific states and circumstances [25]. To get deeper insights into the mechanisms modulating the p53 response, we focused on its interaction with the NF-κB network, a key pathway for regulating cell survival and immunity [26]. The NF-κB network is activated by extracellular ligands or intrinsic stress signals. Extracellular ligands like TNFα bind to specific trans-membrane receptors, leading to activation of the trimeric IKK complex. The kinase IKK2 then phosphorylates IκBα which is an inhibitor of NF-κB transcription factors [27]. Upon phosphorylation, IκB is ubiquitinated and degraded by the proteasome. As a consequence, NF-κB transcription factors enter the nucleus and activate transcription of cellular response genes [28,29]. In addition to genes required for cell survival and proliferation, these response genes include signaling molecules such as IL-6 that activate further downstream signaling pathways in an auto- or paracrine manner [30,31]. Interestingly, NF-κB can also be activated by ATM in response to genotoxic stress [32,33]. This DNA damage-dependent NF-κB signaling represents a first important link between p53 and NF-κB.

There is evidence for several interactions between both networks mediated primarily through IKK2. It has been shown that IKK2 phosphorylates p53 and promotes its degradation upon DNA damage independently from Mdm2 via the SCF complex [34]. Inhibiting IKK2 leads to increased p53 accumulation and acetylation, expression of the cell cycle inhibitor p21, an important p53 target gene, and induction of apoptosis [35]. Other reports indicate that NF-κB may have a stabilizing function on p53 [36].

Another important point of crosstalk may be the phosphatase Wip1, which was reported as transcriptional target of NF-κB in breast cancer cells. Moreover, its mRNA and protein levels are influenced by NF-κB activation or inhibition [37]. Finally, downstream pathways such as IL-6-induced STAT3 signaling can interfere with p53 signaling as well, for example by modulating the expression level of the tumor suppressor [38].

Given the extensive and partially contradicting nature of reported network interactions, we aimed to systematically investigate how the complex interplay between both signaling pathways determines the cellular response to genotoxic stress. Due to the fundamental impact of p53 dynamics on cell fate decisions, we aimed for a time-resolved understanding of this crosstalk. To this end, we combined quantitative time-lapse imaging of hundreds of individual cells with subpopulation-based mathematical modeling of the underlying molecular network. Using this approach, we could show that perturbations of the IKK/ NF-κB system and its downstream pathways modulate the p53 response to ionizing radiation and alter the subsequent transcriptional response. Sensitivity analysis and parameter inference showed that crosstalk between the IKK/ NF-κB and p53 networks is not restricted to a single interaction but comprises at least two different reactions. We predict potential mechanisms for the modulated p53 response including the activation and degradation of p53 as well as the degradation of Mdm2. The presented framework serves as a blueprint for further systematic analyses of pathway crosstalk by applying targeted network perturbations.

## Results

### Modulation of IKK/ NF-κB signaling alters the p53 response to DSBs

**Fig 1:**
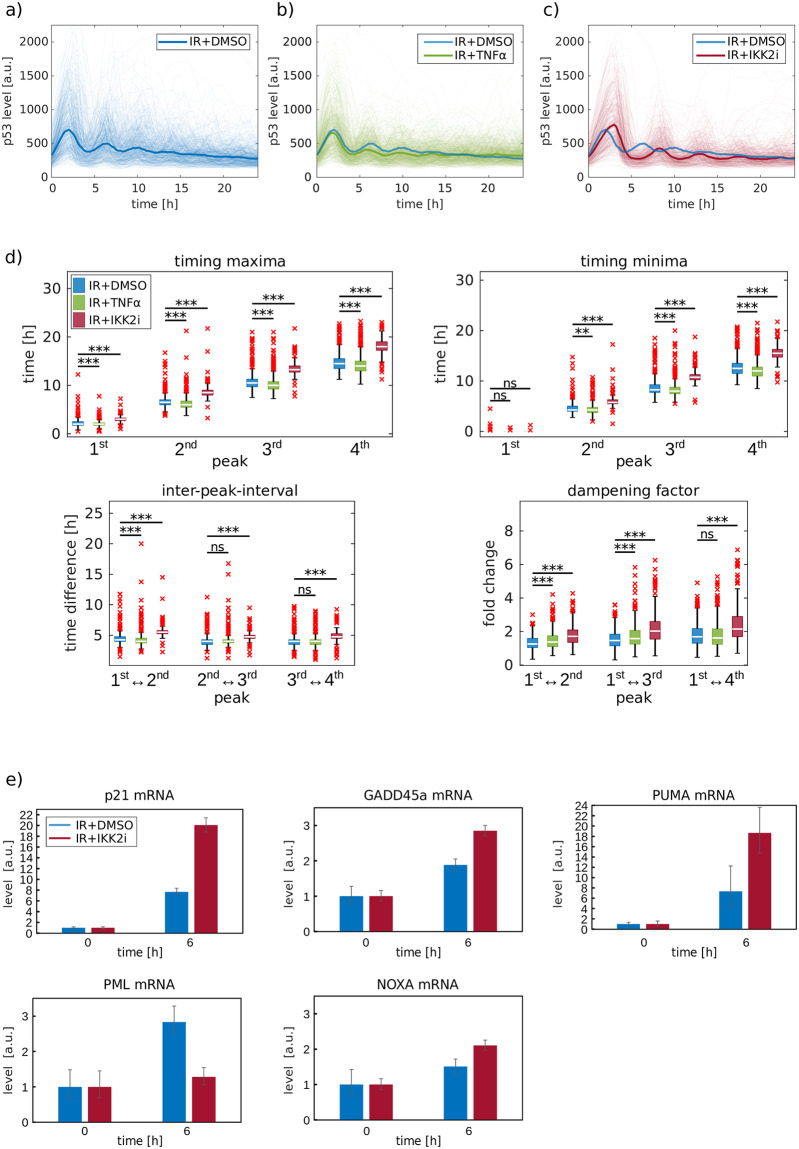
Activation status of NF-κB affects the p53 response to DSBs. A549 reporter cells were tracked via live-cell time-lapse microscopy and the p53 median nuclear fluorescence intensity was measured in cells treated with 10 Gy irradiation (IR) in combination with DMSO (a), TNFα (b) or IKK2i (c). Light colored lines denote single cell trajectories whereas bold lines indicate the median of trajectories. d) Quantification of selected features (timing maxima, timing minima, inter-peak-intervals and dampening factor) of the first four p53 pulses. To test the significance of changes, we used the Wilcoxon rank sum test in combination with the Bonferroni-Holm method to correct for multiple testing, *p<0.05, **p<0.01, ***p<0.001. e) mRNA expression of p53 target genes was measured upon 10 Gy IR in A549 cells treated with DMSO or IKK2i by qRT-PCR. β-actin was used as an internal control. Data were normalized to values of the sample harvested 0 h post IR. Error bars indicate the minimum and the maximum value for the relative quantity of three technical replicates.

To investigate the interplay of p53 and NF-κB signaling, we perturbed the IKK/ NF-κB pathway and monitored the p53 response upon ionizing radiation in individual living cells using time-lapse microscopy. Without modulating NF-κB activity, we observed the expected pulsatile behavior of p53 upon damage induction (control, Fig 1a). When we activated the NF-κB network with TNFα prior to irradiation, we observed only minor changes in p53 dynamics (Fig 1b). However, blocking basal and IR-induced activity of NF-κB by inhibiting IKK2 with the pharmacological inhibitor TPCA-1 (IKK2i, S1a and S1b Fig) [39] prior to irradiation led to a striking delay in the median p53 response across a population of cells (Fig 1c).

To further examine the observed alterations in the p53 response, we defined characteristic features of p53 dynamics (S1c Fig). Quantifying the features for each single cell trajectory revealed that upon TNFα treatment, the maxima and minima of p53 accumulation occurred slightly earlier, while the inter-peak-interval (IPI) between the first peaks tended to be shorter and the dampening of p53 amplitudes higher (Fig 1d). In contrast, IKK2 inhibition led to delayed peak timing and significantly longer inter-peak intervals, compared to the control (Fig 1d). The amplitude of consecutive pulses was also damped. The delayed p53 response in single cells could be robustly observed in biological repeats and was validated on the population level by Western blot analysis (S1f, S1g and S2a Figs). To exclude that addition of TPCA-1 itself is sufficient to induce a p53 response, non-irradiated IKK2i-treated cells were monitored by live cell microscopy. No unspecific p53 response was observed (S2b Fig).

While TPCA-1 is a potent inhibitor of IKK2 activity, it also strongly interferes with STAT3 signaling [40]. To determine whether the observed modulation of p53 dynamics upon treatment with TPCA-1 is caused by altered IKK2 or STAT3 activity, we tested two structurally unrelated IKK2 inhibitors for their influence on the p53 response to genotoxic stress. Importantly, all applied inhibitors caused a delay in p53 dynamics (S2c Fig). In particular, timing of peak maxima and minima, IPI, as well as dampening of peaks were consistently altered. Other features such as peak amplitude were differentially effected by the inhibitors (S2d Fig).

As only TPCA-1 affected STAT3 signaling and activation of STAT3 by IL-6 did not alter the p53 response to genotoxic stress (S2e and S2f Fig), we assumed that the observed changes in p53 dynamics upon TPCA-1 treatment were mainly based on IKK2 inhibition.

Importantly, these altered p53 dynamics changed the cellular response to damage as we measured altered expression profiles of p53 target genes involved in cell cycle arrest (p21), damage repair (GADD45), apoptosis induction (PUMA, NOXA) or senescence (PML) (Fig 1e).

### Reproducing the p53 response to DSBs using subpopulation-based modeling

To identify the molecular interactions connecting both networks and driving the observed changes in p53 dynamics, we employed mathematical modeling based on ordinary differential equations (ODEs). ODE models allow to assess the impact of individual model parameters on the behavior of the entire network. Here, we aimed to link altered p53 dynamics to defined parameter perturbations and thereby predict the processes affected by IKK2 inhibition. This requires a model that is capable of faithfully reproducing the time-resolved p53 response to ionizing radiation. As the p53 response among single cells is heterogeneous (Fig 1a) and we needed to analyze quantitative changes that may be masked in population averages, the model should be able to account for the cellular heterogeneity. We therefore developed a framework for subpopulation-based modeling derived from the approach of Strasen et al. [41]. In this approach, single cell trajectories with similar dynamics are clustered into subpopulations and averaged. Note that the aim of our clustering approach is to capture the heterogeneity of the individual cell dynamics in a given experiment rather than describing different types of dynamics. In order to reproduce all subpopulation dynamics, a pool of models is generated in which each model is fitted to the averaged trajectories.

**Fig 2:**
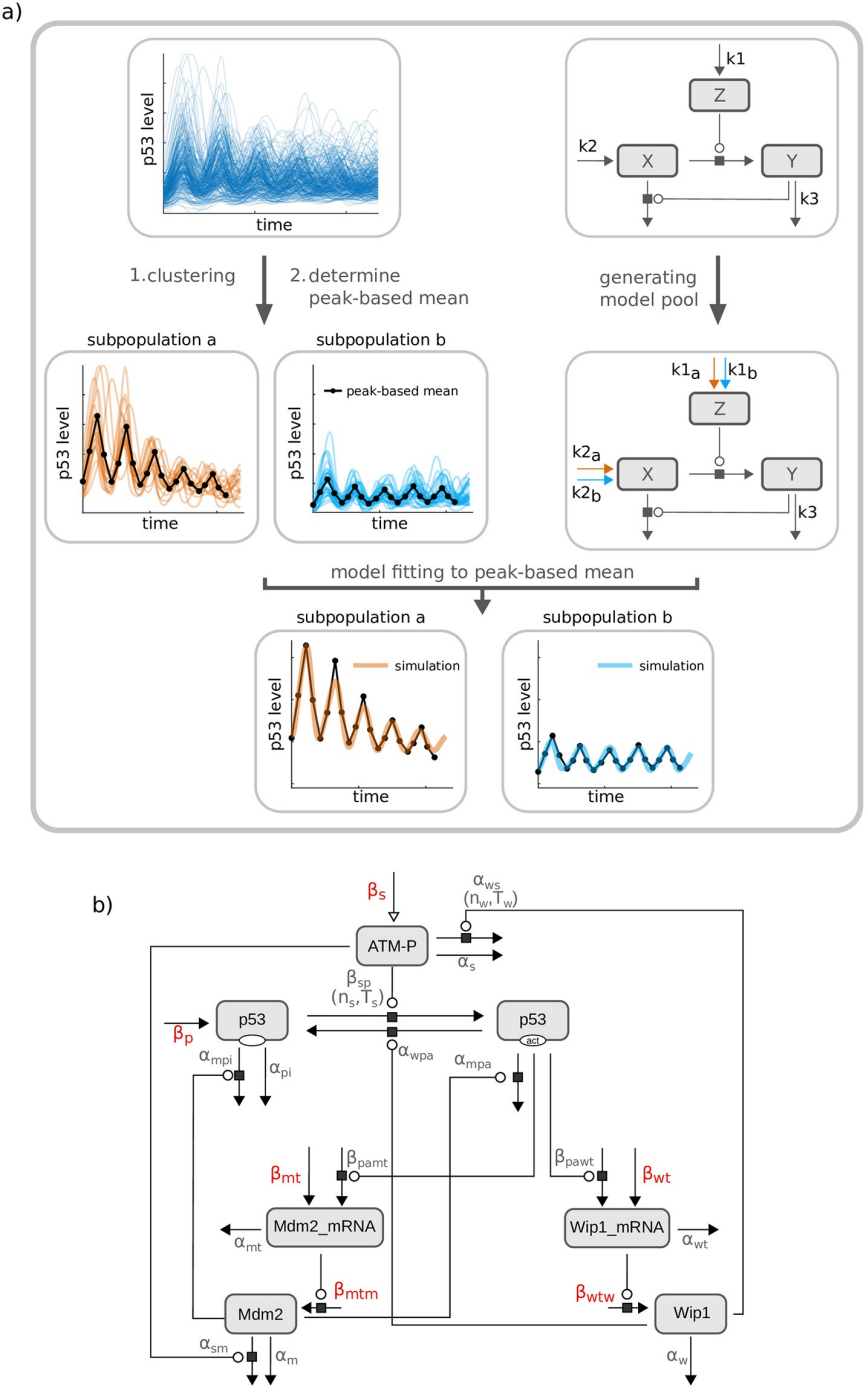
Scheme of the subpopulation modeling framework and the model describing the p53 network. a) Single cell trajectories are clustered into subpopulations with similar dynamics (upper left panel). For each cluster, a peak-based mean is calculated, representing subpopulation-specific dynamics. To account for cellular heterogeneity and to reproduce the different dynamics of subpopulations, a pool of models is generated (upper right panel). In this example, the model pool comprises two models, one is specific for subpopulation *a*, the other one for subpopulation *b*. The production rates of *Z* and *X* are assumed to be susceptible to noise. Hence, parameters of these two processes are considered subpopulation-specific and therefore specific for an individual model. While *k1*_*a*_ and *k2*_*a*_ are specific for subpopulation *a* and thus assigned to one model, *k1*_*b*_ and *k2*_*b*_ are specific for subpopulation *b* and assigned to the second model. Importantly, both models share parameters such as *k3*, assuming that for example phosphorylation rates or degradation rates are not affected by noise. In a final step, the model pool is simultaneously fitted to the peak-based mean of each subpopulation. b) The ODE model consists of seven variables representing phosphorylated ATM (ATM-P), p53 in its inactive and transcriptionally active (act) form, as well as mRNAs and proteins of Mdm2 and Wip1. Moreover, it contains 22 parameters; those marked in red are defined as subpopulation-specific since the corresponding processes are assumed to be susceptible to noise.

We applied this approach to our single cell data by establishing a framework for clustering p53 dynamics into subpopulations. To preserve the pulsatile characteristics of single cell trajectories we averaged the trajectories within a subpopulation in a peak-wise manner (peak-based mean) by determining mean timings and mean absolute values of peaks (Fig 2a, see S1 Text for details.) To reproduce these dynamics, we adjusted an initial model derived from Batchelor et al. (Fig 2b, [42]) by introducing subpopulation-specific parameters. This model describes the p53 response to DNA damage and contains the two feedback regulators Mdm2 and Wip1 as well as the kinase ATM. We extended the model by including variables for Mdm2 mRNA and Wip1 mRNA with corresponding production and degradation rates (see S1 Text). For the definition of subpopulation-specific parameters, we focused on parameters affecting production rates of mRNAs and proteins, as these are susceptible to intrinsic noise [20,43,44]. In contrast, parameters accounting for biochemical processes such as phosphorylation reactions remained constant across the model pool (Fig 2b).

**Fig 3:**
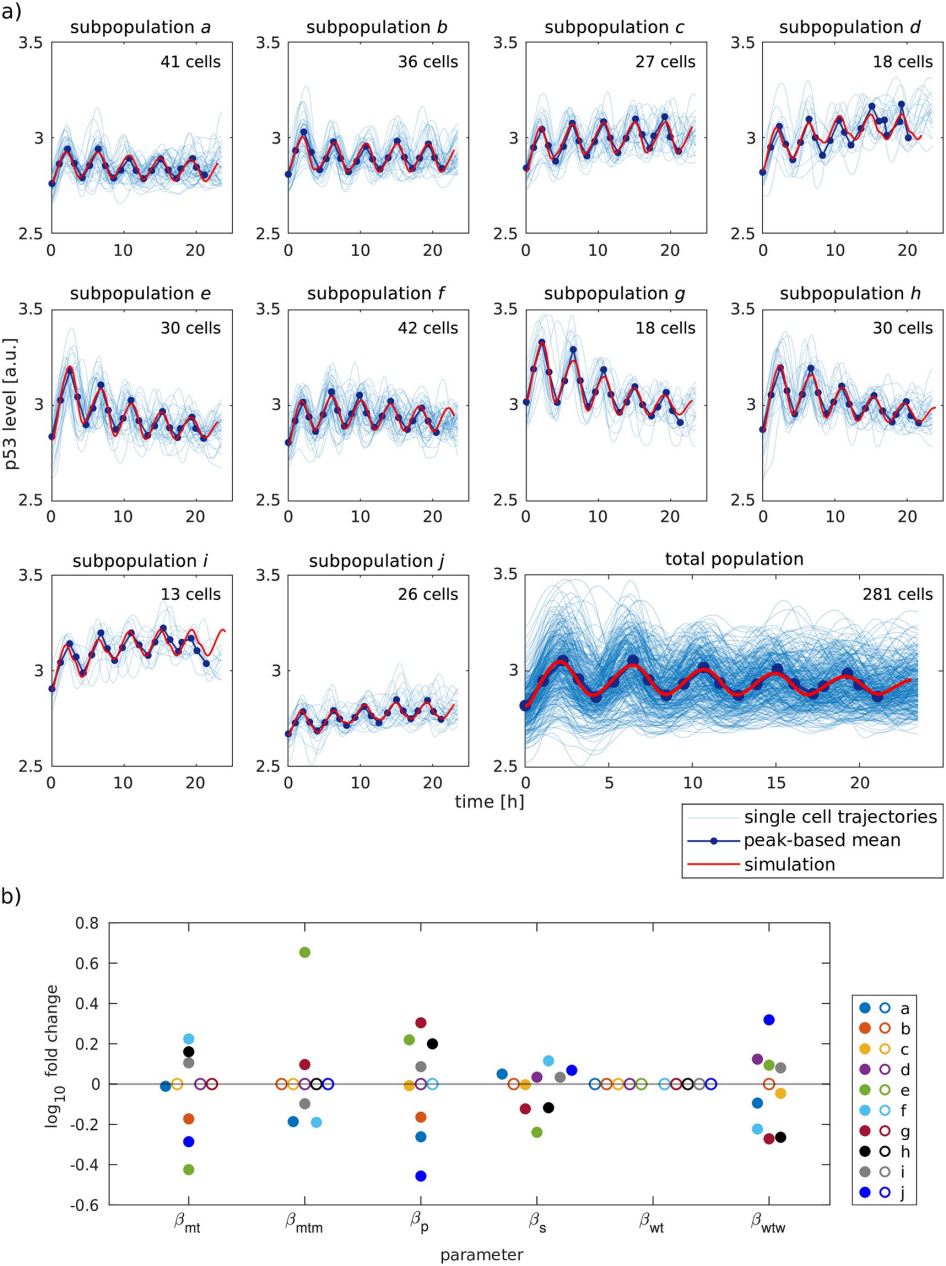
Model pool reproduces heterogeneous p53 dynamics. a) The ten models of the model pool were fitted to the peak-based mean of the ten subpopulations (blue lines). The red line indicates the simulation of the best fit. The number of cells assigned to a subpopulation is stated in the upper right corner of each subpopulation plot. To visualize the simulation and experimental data on the population level (lower right corner), the weighted mean over all subpopulations was calculated. The weight is determined by the number of assigned cells. b) L1 regularization-based evaluation of subpopulation-specific parameters. The parameters correspond to the six biochemical processes that were assumed to be subpopulation-specific. The processes include basal transcription of the Mdm2 gene (β_mt_), translation of Mdm2 mRNA (β_mtm_), synthesis of p53 (β_p_), activation of ATM (β_s_), basal transcription of the Wip1 gene (β_wt_) and translation of Wip1 mRNA (β_wtw_). Subpopulation-specific parameters are represented by colored dots. Each color denotes an individual subpopulation. Parameters with a value close to zero are identified by L1 regularization as unspecific (circles).

We applied our framework to single cell data capturing the p53 response upon irradiation (calibration data) by determining pairwise dissimilarities between trajectories, subjecting the dissimilarities to a hierarchical clustering algorithm and calculating the Calinski-Harabasz index to identify the number of clusters in the data set (see S1 Text for details). This analysis suggested to employ ten subpopulations to represent the calibration data set (Fig 3a, blue lines).

Note, that these subpopulations do not reflect distinct physiologically states. Instead, we cluster trajectories with similar dynamics into subpopulations in order to reflect the variability of p53 dynamics observed in the population due to cellular heterogeneity.

Next, we calibrated the model pool by fitting models simultaneously to the peak-based mean of each subpopulation. To refine the subpopulation-specific parameters and identify those parameters driving heterogeneity, we employed L1 regularization [45] (see S1 Text for details). The regularization is applied to model parameters during optimization and allows to identify parameters that are negligible for reproducing the data. With this approach, we reduced the number of subpopulation-specific parameters from 60 (10 subpopulations with 6 parameters each) to 38 and generated minimal models sufficient to describe the variance among subpopulation dynamics (Fig 3a, red lines).

Comparison of the subpopulation-specific parameters illustrates the quantitative differences between subpopulations that are not necessarily apparent from the dynamics (Fig 3b). Interestingly, subpopulation-specific fold changes of parameters in the range of -0.46 and +0.65 (on a log10 scale) are sufficient to reproduce the heterogeneity between the subpopulations. Of note, basal transcription of the Wip1 gene (β_wt_) was identified as unspecific and is therefore not necessary to reproduce the variability in p53 dynamics. Averaging all subpopulation simulations again reproduced the average p53 dynamics of the cell population (Fig 3a).

Taken together, by clustering single cell trajectories of p53 into ten subpopulations and fitting a pool of ten ODE models to the peak-based representation of subpopulation dynamics, we were able to quantitatively reproduce the dynamic response of p53 to DSBs.

### IKK2 is predicted to act on multiple processes of the p53 network

To link the observed changes in p53 dynamics upon IKK2 inhibition to molecular processes within the network, we performed a sensitivity analysis and compared changes induced by parameter perturbations in the model to our experimental measurements. We focused on the four features of p53 dynamics that were previously shown to be significantly and consistently increased upon inhibition of IKK2 with different inhibitors (Fig 1d, S2d Fig), including timing of pulse maxima and minima, IPI as well as dampening of pulses. For the sensitivity analysis, the 22 kinetic parameters shared among all models were perturbed individually and the effect on the four features was quantified in a peak-wise manner across subpopulations (S3 Fig). To simplify the comparison between the results of the sensitivity analysis and the experimental data, we condensed the peak-wise sensitivity coefficients to a single value using defined threshold values (see S1 Text for details).

**Fig 4:**
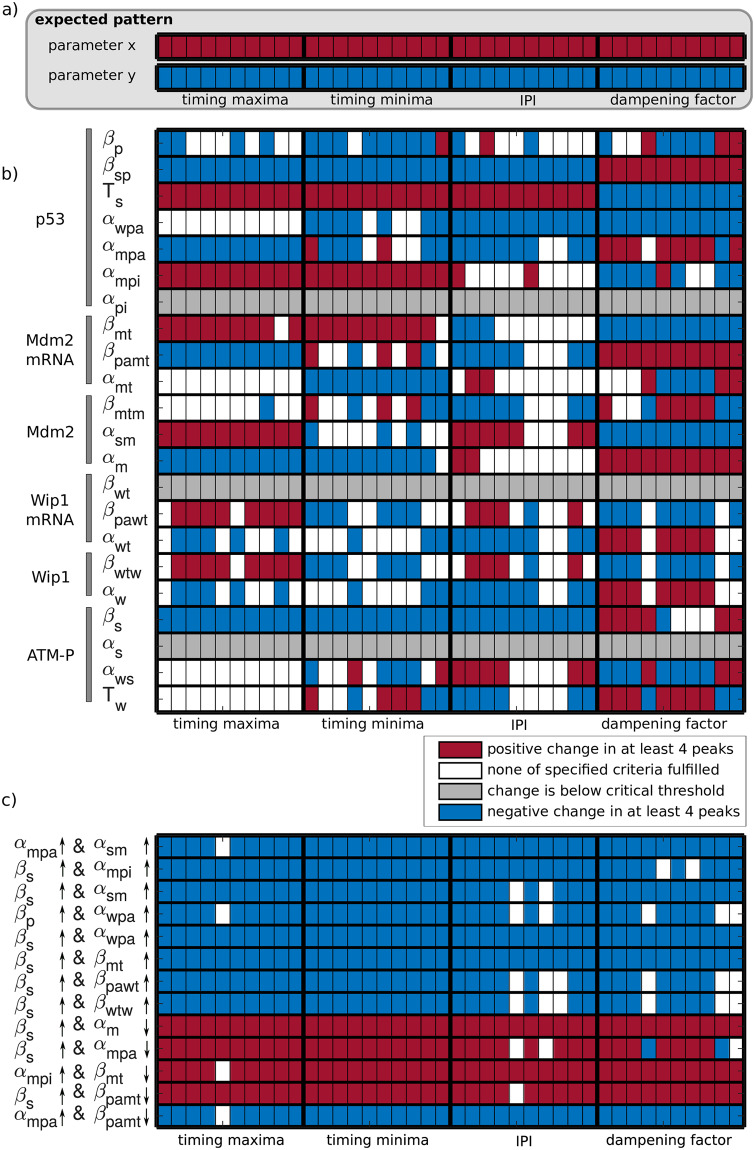
Sensitivity analysis predicts existence of multiple interactions of IKK2 and the p53 network. a) Expected change in features upon perturbation of a parameter, which indicates an interaction of IKK2 and the p53 network. Each box represents the effect of a parameter perturbation on a specified feature for an individual subpopulation. A red box indicates a consistent increase in the specified feature, in all considered peaks. Blue boxes represent a decrease. b) Sensitivity analysis of single parameters. If the absolute value of sensitivity coefficients of all considered peaks are below 1·10^−4^, the specified parameter change has no considerable effect on the respective feature (grey boxes). Subpopulations with sensitivity coefficients of peaks which are not consistently changed are depicted in white. c) For the sensitivity analysis of parameter pairs, 13 pairs were selected from 462 possible combinations based on their ability to reflect the expected change in features in at least seven out of ten subpopulations. Arrows indicate a perturbation of parameters by increasing or decreasing the respective parameter value by 1%.

The experimental observations lead to the ‘expected pattern’ (Fig 4a): perturbation of a parameter should lead to a consistent change, that is either an increase or a decrease, in the features timing of maxima, timing of minima, IPI, and dampening across subpopulations. We perturbed parameters by +1% and considered both an increase and a decrease in all feature values, as IKK2 may exert positive or negative influence on a given process. However, none of the model parameters fulfilled this criterion (Fig 4b). At least one feature showed contradicting effects in one or all subpopulations. Moreover, the parameters for basal degradation of p53 (α_pi_), basal degradation of ATM-P (α_s_) and basal transcription of the Wip1 gene (β_wt_) showed no considerable effect on any feature. To assess if a stronger or negative perturbation of parameters would compromise these results, we performed additional sensitivity analyses in which we perturbed parameters by +30% and -30%, respectively (S4 and S5 Figs). Note, that even stronger perturbations greatly affect pulsatile dynamics and renders quantification of features infeasible.

The result of the sensitivity analysis showed that none of the single parameter perturbations lead to the ‘expected pattern’ and therefore strongly indicated that modulation of the p53 response by IKK2 inhibition is due to multiple interactions between IKK2 and the p53 pathway. We therefore hypothesized that simultaneous perturbation of two parameters might reproduce the observed changes in all four features. To test this, we performed a sensitivity analysis on all possible pairwise combinations of parameters. As it is not known if IKK2 inhibition has the same amplifying or attenuating effect on two processes, the sign of the perturbation can be different for two parameters. We therefore performed the sensitivity analysis by increasing both parameters or increasing one parameter and decreasing the second one. Using this approach, we identified 13 out of 462 possible combinations that are able to reflect the consistently observed effects of IKK2 inhibition (Fig 4c).

### Parameter inference-based predictions of interactions between IKK2 and the p53 network

Although the sensitivity analysis enabled us to make predictions about putative interaction points of the NF-κB and p53 pathway, the analysis does not cover different perturbation strengths of parameters or the extent to which individual features are changed. By taking additional quantitative information into account, we sought to refine the identification of potential interaction points in two ways. First, evaluating different perturbation strengths would enable us to capture additive and compensatory effects in a more comprehensive way. As a consequence, additional parameter combinations might be revealed which would not have been identified by fixed perturbation strengths. Second, taking quantitative information into account provides more stringency, as parameter combinations reflecting feature changes in a qualitative manner might not be able to reflect changes quantitatively. Therefore, those parameter combinations could be excluded as potential targets of the crosstalk between the NF-κB and p53 pathway.

Hence, in a second approach, we employed parameter inference to include quantitative changes in dynamics and derive perturbation strengths from experimental data by fitting individual parameters of the calibrated model pool to trajectories from IKK2i-treated cells (perturbation data). All remaining parameters were fixed to their calibrated value. This allowed us to evaluate parameters with respect to their capability to shift p53 dynamics upon DSBs to the altered dynamics of p53 upon IKK2 inhibition. As we assume that inhibition of IKK2 affects the same process with the same magnitude in all subpopulations, we only fitted parameters that are shared among models of the model pool.

In order to fit those parameters to the perturbation data, trajectories of the perturbation data had to be assigned to the ten clusters of the calibration data. The assignment is based on the assumptions that inhibition of IKK2 i) leads to the same qualitative changes in dynamics in all subpopulations and ii) does not affect the composition of subpopulations. For the assignment, we defined nine criteria (Table B in S1 Text) that we derived from experimentally observed changes in features upon IKK2 inhibition. Based on the criteria we assigned individual trajectories from the perturbation data to one of the ten clusters (S1 Text). In order to be able to fit the model pool to the perturbation data, the peak-based mean of the assigned trajectories was determined for each subpopulation (S6a Fig) and compared to the calibration data (S6b Fig). The results are discussed in S1 Text.

As our previous sensitivity analysis indicated that multiple interactions points exist between IKK2 and the p53 network, we tested if simultaneous fitting of two parameters allows to quantitatively reproduce the modulated p53 response. To this end, all possible combinations of parameter pairs were fitted to the perturbation data, while the remaining parameters were fixed to their calibrated value. However, the model pool was unable to reproduce the observed pulsatile dynamics of p53 with any of the fitted parameter pairs as demonstrated by the parameter combination with the best fit quality (S7a Fig). We therefore expanded our approach to parameter triplets and identified several combinations that yielded improved fit qualities (S7b Fig). The model pool with the best fitted parameter combination successfully reproduced the altered p53 response to DSBs upon IKK2 inhibition (S7c Fig, left), demonstrating that perturbation of three parameters is sufficient to capture the effect of IKK2 on the p53 network. As parameter combinations differ in their corresponding fit qualities (S7c Fig), the performed parameter inference allows to distinguish parameter combinations based on their capability to reproduce a targeted perturbation. This indicates that the fit quality can be used to predict putative interfaces of the NF-κB and p53 pathway.

### Validation of predicted parameter combinations reveals two potential mechanisms for the observed alterations in p53 dynamics

To validate the best ranked parameter combinations, we compared parameter inference-based predictions with additional experimental observations. We tested the 30 best ranked parameter combinations to gain a broader overview of model performance (S8 Fig).

**Fig 5:**
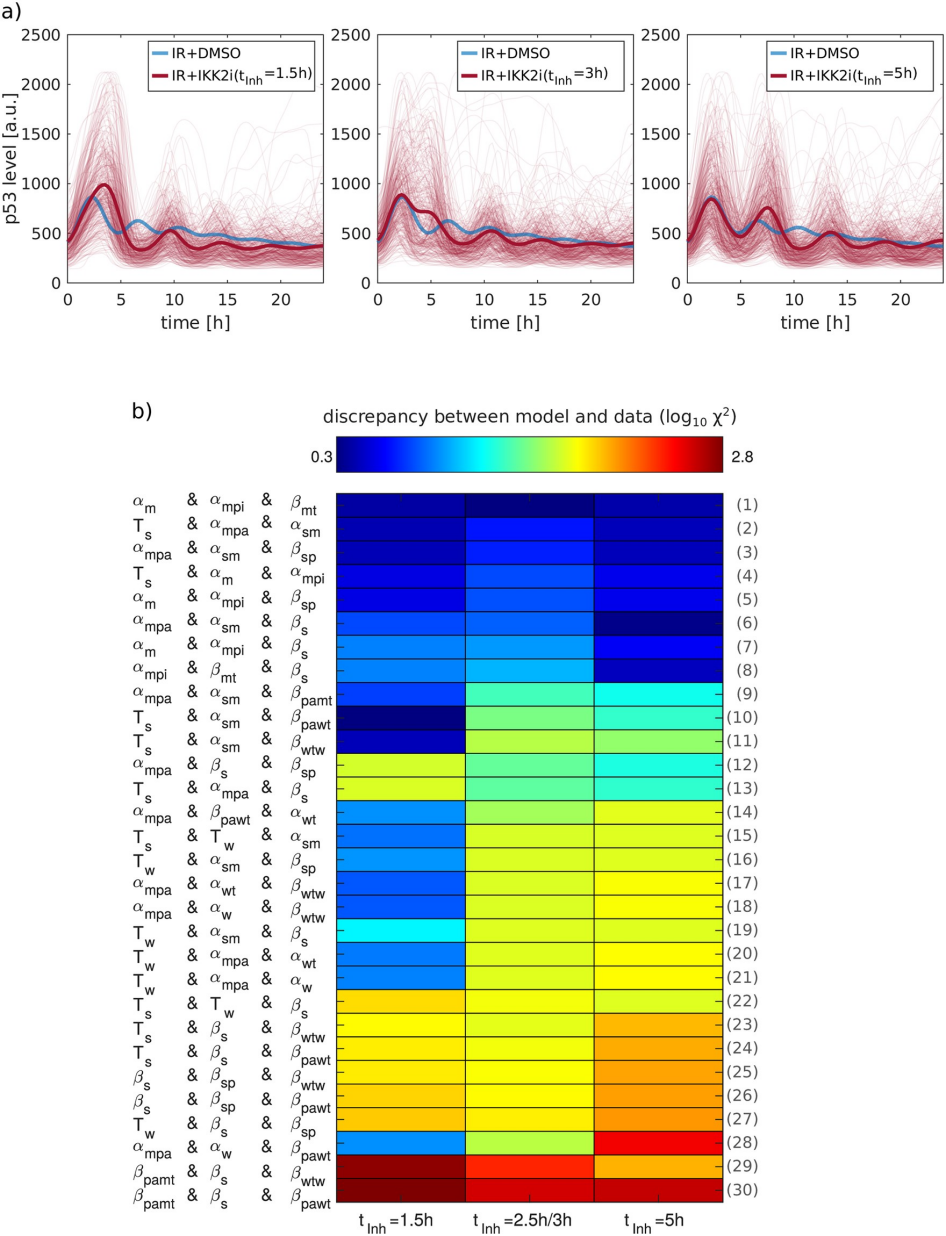
Validation of the 30 best ranked parameter combination fits using time-variant IKK2 inhibition. a) A549 reporter cells were tracked and the p53 median nuclear fluorescence intensity was measured in cells treated with DMSO or IKK2i at different time points. Sample trajectories are shown in which IKK2 was inhibited at the indicated time points. In an additional experiment, IKK2 was inhibited at 1.5h, 2.5h and 5h after IR. b) Each box depicts the color coded log_10_ χ^2^ value for model simulations with individual parameter combinations and time points of IKK2 inhibition. For better visualization, the plotted log_10_ χ^2^ value summarizes the values of two data sets with similar time points of IKK2 inhibition (1.5h/1.5h, 2.5h/3h, 5h/5h). The inhibitor was applied at accumulating levels of p53, resulting in the first peak (t_Inh_ = 1.5h), decreasing p53 levels (t_Inh_ = 2.5h and t_Inh_ = 3h) as well as at the accumulation resulting in the second peak (t_Inh_ = 5h). The parameter combinations are sorted based on the corresponding summarized log_10_ χ^2^ value. Indices of the parameter combinations are given by the numbers on the right hand side of the plot.

As additional perturbations, we used time-resolved single cell data, where the IKK2 inhibitor TPCA-1 was applied at different time points post damage (1.5h, 2.5h/3h, 5h, Fig 5a). To systematically evaluate parameter combinations, the discrepancy between model simulations and experimental data was determined by calculating the weighted χ^2^ value for each parameter combination and each data set (see S1 Text for details). Eight parameter combinations were able to reproduce p53 dynamics for all perturbation times with only small deviations (Fig 5b, top 8 parameter combinations).

In order to visualize the difference between the eight parameter combinations and the remaining combinations, model simulations with selected parameter combinations were compared (S9 Fig). While the p53 dynamics upon IKK2 inhibition 1.5h post damage could be reflected by all selected parameter combinations, the 2.5h and 3h time points of IKK2 inhibition were qualitatively less well reflected by the combinations. The strongest difference between the three combinations selected from the best eight parameter combinations (α_m_ & α_mpi_ & β_mt_, T_s_ & α_m_ & α_mpi_, α_mpi_ & β_mt_ & β_s_) and the ninth parameter combination (α_mpa_ & α_sm_ & β_pamt_) was observed when the IKK2 inhibitor was applied five hours after irradiation. While the three combinations reflected the continuous accumulation of p53 observed in the data, the combination α_mpa_ & α_sm_ & β_pamt_ showed a sudden and strong decrease in p53 levels. Thus, we selected the eight parameter combinations with the smallest χ^2^ values in all data sets of time-variant IKK2 inhibition for further validation.

**Fig 6:**
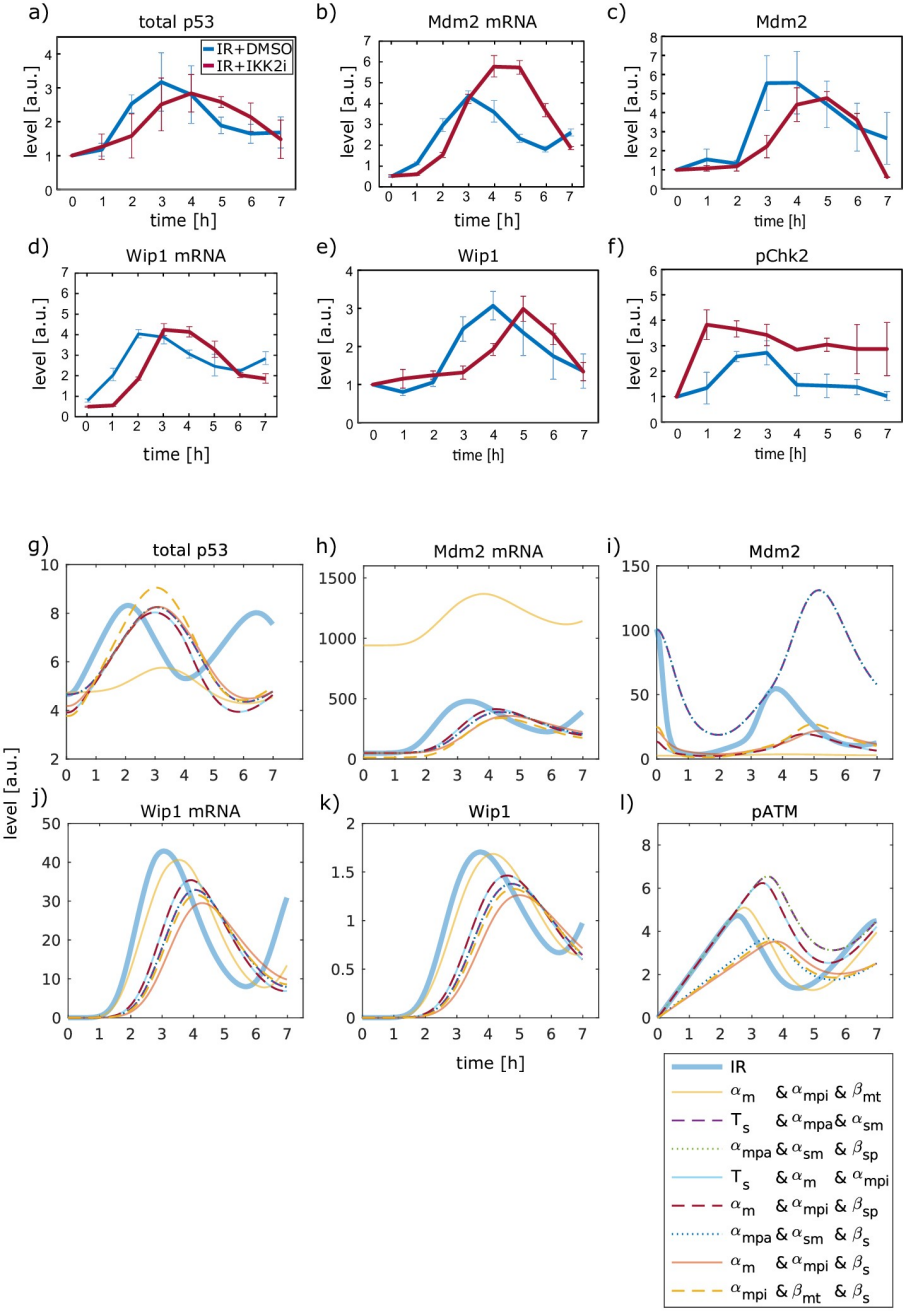
IKK2 inhibition leads to a delayed Wip1 and Mdm2 accumulation and increased pChk2 levels on the population level. Upon 10 Gy IR in A549 cells treated with DMSO or IKK2i, the levels of p53 (a), Mdm2 mRNA (b), Mdm2 protein (c), Wip1 mRNA (d), Wip1 protein (e) and pChk2 (f) were measured and quantified. Protein levels were determined by Western blot analysis. Data from independent experiments were normalized to values of the sample harvested 0 h post IR. Error bars indicate standard deviation of two biological replicates. mRNA expression was measured using qRT-PCR. β-actin was used as an internal control. Error bars indicate the minimum and the maximum value for the relative quantity of two biological replicates, each consisting of three technical replicates. Simulated dynamics of total p53 (g), Mdm2 mRNA (h), Mdm2 (i), Wip1 mRNA (j), Wip1 (k) and pATM (l) are shown for eight parameter combinations and IR alone.

For additional validation, we used Western blot and qRT-PCR data to analyze the effect of IKK2 inhibition on dynamics of additional components of the p53 network. Based on the described delay in the p53 response (Fig 6a and S2a Fig), the impact on the two negative feedback regulators Mdm2 and Wip1 was tested first. We observed that Mdm2 as well as Wip1 protein accumulation was delayed upon IKK2 inhibition (Fig 6c and 6e and S10a Fig). Moreover, IKK2i treatment resulted in a delayed increase of Mdm2 and Wip1 mRNA levels as well, indicating that gene expression of both feedback regulators followed altered p53 dynamics as expected (Fig 6b and 6d). To determine ATM activity upon perturbation, we measured the levels of one of its substrates, phosphorylated Chk2, over time. Surprisingly, we detected higher phosphorylation levels upon IKK2i treatment (Fig 6f and S10b Fig).

When we compared the simulated dynamics for the previously mentioned components with the experimental data, we noticed for all eight parameter combinations a similarly delayed accumulation of Mdm2 and Wip1 on the mRNA level as well as on the protein level (Fig 6h–6k). However, the best ranked parameter combination from the previous validation in which Mdm2-mediated degradation of p53 (α_mpi_), basal degradation of Mdm2 (α_m_) and basal transcription of the Mdm2 gene (β_mt_) were perturbed, predicted very high levels of Mdm2 mRNA, and very low Mdm2 protein levels (Fig 6h and 6i). This is not in line with experimental observations (Fig 6b and 6c). Moreover, the parameter combinations in which the activation of ATM is affected (β_s_) showed a decreased kinase activity and thus failed to reflect the experimental data (Fig 6l and 6f) as well. Those parameter combinations were therefore not considered further. Note, that the dynamics of Mdm2 on the protein level within the first hour differ between simulation (Fig 6i) and experimental data (Fig 6c) as we did not include the population data into model calibration. Interestingly, such an initial decrease in Mdm2 levels was also observed experimentally [19,42].

**Fig 7:**
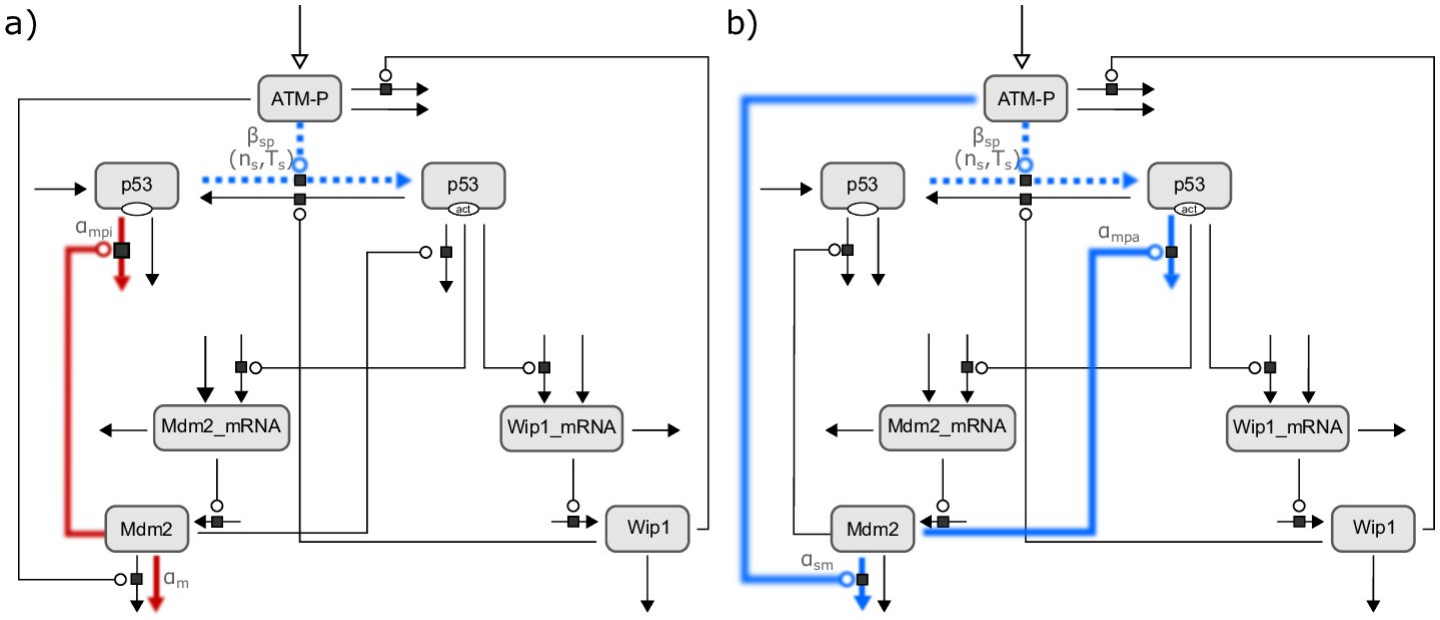
Scheme of the two identified mechanisms allowing to reflect the effects of IKK2 inhibition on p53 dynamics. a) The first mechanism comprises the two parameter combinations α_mpi_ & α_m_ & β_sp_ and α_mpi_ & α_m_ & T_s_. The dotted lines indicate that the parameter combinations differ in the corresponding parameters (β_sp_ and T_s_), but the affected process is the same. In both parameter combinations the process is predicted to be reduced, which is denoted by the blue color. In contrast, the red solid lines represent the two processes shared among the two combinations, which are predicted to be increased. b) The parameter combinations α_mpa_ & α_sm_ & β_sp_ and α_mpa_ & α_sm_ & T_s_ constitute the second mechanism. In accordance to the first mechanism, the two parameter combinations differ in parameter β_sp_ and T_s_. The two parameters affecting ATM-induced degradation of Mdm2 (α_sm_) and Mdm2-mediated degradation of activated p53 (α_mpa_) are predicted to be reduced upon IKK2 inhibition.

Finally, four parameter combinations remained, which were able to reflect the data of the second validation. Analyzing the composition of these parameter combinations, unravels two mechanisms explaining the effect of IKK2 inhibition on p53 dynamics (Fig 7). On the one hand, Mdm2-mediated degradation of inactive p53 and basal degradation of Mdm2 could be increased (Fig 7a). On the other hand, ATM-mediated degradation of Mdm2 and Mdm2-mediated degradation of active p53 could be decreased (Fig 7b). Of note, the latter combination was also one of the predicted parameter combinations of the sensitivity analysis. Both mechanisms share a reduced activation rate of p53. These results strongly indicate that the NF-κB and p53 networks interact at multiple independent nodes and provide starting points for further experimental investigation.

## Discussion

Crosstalks between cellular signaling pathways are essential for integrating external and internal inputs. In our approach we studied crosstalk mechanisms by capturing the effect of targeted perturbations of a selected signaling network on the response of an interacting pathway. Combining time-resolved single-cell measurements with subpopulation-based modeling enabled us to systematically analyze such a crosstalk and predict molecular interaction points. Using two central signaling networks of the human stress response as paradigm, we experimentally observed that dynamics of p53 upon DNA damage were affected by the activity state of the NF-κB network. Our corresponding model-based analysis indicated that none of the previously considered network interactions alone were sufficient to explain the observed modulation of p53 dynamics. Instead, we predict that multiple parallel interactions between both networks shape the signaling response and suggest possible molecular mechanisms for further validation.

With our approach, we predicted two possible mechanisms how the p53 network is modulated by the applied pharmacological perturbations. For each mechanism, three processes are affected: degradation of inactive or active p53, degradation of Mdm2 as well as activation of p53. Although a further confinement of our predictions was not possible, a number of studies exist that support the predictions and provide a molecular basis for our proposed interaction with the IKK/ NF-κB network. IKK2 was shown to interfere with the degradation of p53 [34,35]. Inhibiting IKK2 activity resulted in both studies in a decreased degradation rate of p53 which is in line with our prediction that inhibition of IKK2 reduces the degradation of active p53 (α_mpa_). However, the degradation of p53 was reported to be independent of Mdm2 which is not in agreement with our prediction [34]. More recently, Ishak Gabra et al. uncovered that IKK2 phosphorylates p53 at serine residue S392 [46], which was previously shown to affect p53 degradation as well as its DNA binding and tetramerization [47–50]. Preventing phosphorylation of p53 at S392 resulted in a reduced degradation of p53 [49,50]. Interestingly, Du et al. could link the degradation to ubiquitination of p53 by the ubiquitin ligase UBE4B [50], which is known to promote Mdm2-mediated degradation of p53 [51]. Thus, one can hypothesize that IKK2 inhibition reduces the Mdm2-induced degradation of p53 by preventing its ubiquitination by UBE4B.

Another prediction of the model was that IKK2 affects degradation of Mdm2. In particular, inhibition of IKK2 was predicted to either increase the basal degradation of Mdm2 (α_m_) or reduce the ATM-dependent degradation of Mdm2 (α_sm_). Indeed, IKK2 can phosphorylate Mdm2 at S166 [52]. A previous study has indicated that inhibition of Akt-mediated phosphorylation of Mdm2 at this serine residue prevented translocation of Mdm2 to the nucleus and caused increased p53 levels [53]. Consequently, it is possible that inhibition of IKK2 decreases the amount of Mdm2 that is available in the nucleus for the degradation of p53. Reduced levels of Mdm2 upon IKK2 knockout [54] support this notion and favor our predicted increase in the degradation of Mdm2 (α_m_). Conversely, preventing the activation of NF-κB by overexpression of a non-degradable IκBα mutant led to increased levels of Mdm2 [36]. As IKK2 inhibition prevents activation of NF-κB, the latter result supports our predicted reduction in the degradation of Mdm2 (α_sm_).

Moreover, the model predicted a reduced ATM-mediated activation of p53 upon IKK2 inhibition. This is in line with results of Ishak Gabra et al. who analyzed the phosphorylation of p53 at S15 and S392. Phosphorylation of p53 at S15 is known to be mediated by ATM [55]. Blocking the phosphorylation of p53 at S392 resulted in delayed phosphorylation of p53 at S15 [56]. These results indicate that IKK2 could indeed reduce ATM-mediated activation of p53.

Taken together, these experimental observations corroborate most of our predicted interaction points between the IKK/ NF-κB and p53 network (S10c Fig). However, we can currently not fully exclude further contributions from other signaling pathways. TPCA-1 is a known inhibitor of STAT3 signaling, which we verified in our experimental system. While the other IKK2 inhibitors used in this study did not directly block STAT3 phosphorylation, they may indirectly alter STAT3 activity due to reduced expression of activating ligands such as IL-6 upon loss of NF-κB activity in damaged cells. STAT3 was reported to regulate p53 expression by binding to its promoter [38]. As the fluorescent p53 reporter is expressed from a constitutive promoter, it is however unlikely that this proposed interaction caused the observed changes in p53 dynamics.

While the detailed molecular interactions need to be further analyzed in future studies, our systematic approach clearly indicates that the networks employ several interaction points simultaneously.

Our approach is based on three key features: selecting suitable molecular perturbations, obtaining time-resolved quantitative measurements and devising an appropriate modeling strategy. While the proposed approach can be used as a blueprint to investigate crosstalk in other signaling systems the features need systems-specific consideration. For the molecular perturbations, we here used characterized ligands and pharmacological inhibitors to perturb cellular signaling. Pharmacological inhibitors have the advantage that they act almost immediately upon treatment, providing flexibility for time-variant perturbations and little opportunity for a network to compensate the loss of essential activities [57]. However, small-molecule inhibitors are often restricted by limited specificity, leading to off-target effects that can hinder the interpretation of a given perturbation [58]. We addressed this constrain by employing multiple structurally independent inhibitors and focusing on features of dynamics that were consistently altered by all molecules. Moreover, most pharmacological inhibitors target signaling proteins with enzymatic functions, mainly kinases, which restricts the network nodes amenable to perturbations. Alternative strategies would rely on genetic perturbations such as siRNA- or shRNA-mediated knockdown or CRISPR/Cas9-mediated knockout of selected network components. However, most of these approaches require extended incubation periods, limiting the precision of perturbation timing and allowing compensation through altered network states.

Another important consideration for designing a perturbation is the position of the targeted network node. Upstream factors provide a broad spectrum of possible interactions. However, their function is often pleiotropic, making it harder to distinguish direct effects on pathway interactions from more indirect effects due to alterations of the cellular state. Conversely, downstream factors provide more defined alterations, but their perturbation may provide a limited scope for analysis. In this study, we focused on the central kinase IKK2, which can affect processes of the p53 network directly or via altered target gene expression.

Upon applying a targeted perturbation, our approach requires time-resolved quantitative measurements to characterize the resulting alterations in the interacting signaling pathway. Therefore, we applied time-resolved live-cell imaging of fluorescent reporter cell lines as a non-invasive measurement technique that allows to follow a population of individual cells with high temporal resolution over an extended observation period [59]. The drawback of live-cell imaging is the low number of readouts it provides, limiting the ability for multiplexed measurements. We therefore supplemented the single cell p53 measurement by Western blot and qPCR data for measurements of Mdm2, Wip1 or ATM activity. An alternative approach would be flow cytometry which allows for a higher number of simultaneous read outs, but, due to fixation of cells, lacks information about the read out of individual cells over time [60].

The third challenge is the choice of an appropriate level of model abstraction for successfully analyzing and predicting signaling crosstalk. A model should be compact as computational costs for parameter inference correlate with the dimensions of the model and smaller models are less prone for overfitting. However, it needs to include sufficient detail to provide meaningful entry points for network interactions and to allow predicting the underlying molecular mechanisms. We decided to base our efforts on a previously published model focusing on the feedback structure of the p53 network that shapes its dynamic response [24,61]. We introduced minor changes to make it amenable for our approach while retaining a high level of abstraction. Upon analyzing the general processes affected by network crosstalk, it would now be feasible to expand the model correspondingly and include additional molecular detail along with further experimental validations.

Full reconstruction of cell-to-cell variations on the level of single cell dynamics might be possible by employing nonlinear mixed-effect modeling [62,63]. This approach allows to reproduce single cell trajectories by simultaneous inference of shared and cell-specific parameters. The advantage of such an approach would be that a model-based comparison between different experimental conditions does not require an assignment of trajectories from one condition to the other in contrast to our subpopulation-based approach. However, such mixed-effect modeling approaches are computationally demanding as it requires fitting of the model to each single cell trajectory and is therefore better suited for more confined models. Moreover, it requires a model that is able to capture heterogeneity in the oscillations on the single cell level. Hence, we decided to employ a subpopulation-based approach which reduces the computational costs and facilitates reproduction of pulsatile dynamics of p53 while heterogeneity in dynamics is still reflected. To capture the effect of the inhibitor on the subpopulation level we assigned trajectories from the perturbation data to the clusters of the calibration data using defined criteria and assuming equal effects on subpopulations (see S1 Text for details). To evaluate the impact of IKK2 inhibition on the assignment, we tested whether the percentage of cells from the perturbation data that is assigned to a cluster is in the range of the corresponding percentage of cells from the calibration data. While three subpopulations (a, f and h) show very similar percentages, there are also cases (e.g. subpopulation b) with strong deviations (S6c Fig). Thus, we cannot rule out that we underestimate to a certain extent the effect of the inhibitor on the dynamics. However, based on the assigned cell numbers, subpopulations are weighted for fitting the model pool to the calibration and the perturbation data. Thus, some subpopulations have a higher impact on the fit than others. Comparing the impact of subpopulations on the fit with respect to deviations in the percentages shows that the subpopulations with the smallest deviation in percentages have the highest impact (S6d Fig). Hence, our parameter inference-based approach is less affected by subpopulations with deviating percentages than by subpopulations with similar percentages.

By employing the subpopulation-based approach, we were able to establish, to our knowledge, the first quantitative model capturing heterogeneous dynamics of p53. While our model calibration is based on time-resolved single cell p53 it also reflects the relevant characteristics of Mdm2, Wip1 and ATM dynamics on the population level (Fig 6). However, we did not include experimental data of those dynamics into the model calibration which can lead to uncertainties in estimated parameter values. Therefore, we expect that additional data capturing single cell dynamics of those network components could help to further refine the quantitative model and might improve the inference of the crosstalk mechanism.

We used the quantitative model to identify interactions of the IKK/ NF-κB and p53 network by applying two different approaches. Of note, both approaches, the sensitivity analysis and inference of perturbed model parameters pointed to a similar parameter combination (α_mpa_ & α_sm_) that allows to reflect the perturbation data. The results of the sensitivity analysis are based on experimentally observed changes in features of p53 dynamics upon IKK2 inhibition. As those features were consistently changed by three structurally independent IKK2 inhibitors, the sensitivity analysis provides robust results which are independent of the applied inhibitor and therefore unaffected by off-target effects. In contrast, the parameter inference approach is based on a single data set. Consequently, inhibitor specific effects influence the model-based predictions. However, comparing the effects of the three different inhibitors on the features of p53 dynamics revealed that most of the changes in features are shown by at least two inhibitors (S2d Fig).

Taken together, our approach provides a framework to systematically analyze crosstalk between signaling pathways using perturbation experiments. It can be applied to other systems to get more detailed insights into the highly interconnected network of signaling systems that controls proliferation, cell cycle arrest and apoptosis and therefore the fate decision of every cell in response to stress. Such an understanding will provide the foundation for new strategies for using combinations of targeted pharmacological inhibitors in the treatment of human malignancies such as cancer.

## Material and Methods

### Cells

We cultured A549 cell lines in McCoy’s 5A (GE Healthcare Life Sciences, Freiburg, Germany) plus 10% fetal calf serum (FCS; Thermo Fisher Scientific, Darmstadt, Germany) at 37°C. The medium contained penicillin and streptomycin. For A549 p53-Venus reporter cells [64,65], selective antibiotics (400 μg/ml G418 (Carl Roth, Karlsruhe, Germany) and 50 μg/ml hygromycin (Thermo Fisher Scientific)) were added to maintain transgene expression.

For corresponding treatment of the cells, the medium was replaced with fresh one containing dimethyl sulfoxide (DMSO; Sigma-Aldrich) as a control, IKK2 inhibitor (15 μM TPCA-1, 120 μM sc-514 or 0.93 μM BMS-345541 (MedChemExpress, Sollentuna, Sweden)), 10 ng/ml or 50 ng/ml IL-6 (PeproTech, Hamburg, Germany) or 10 ng/ml TNFα (Enzo Life Science, Lörrach, Germany) 1 h before irradiating cells (if not stated otherwise) with X-rays at a dose rate of 1 Gy/26 s (250 keV, 10 mA).

### Time-lapse microscopy

We seeded cells in poly-d-lysine-coated glass-bottom plates (MatTek, Ashland, MA) or 24 well ibiTreat polymer-bottom plates (ibidi, Martinsried, Germany). The day of the experiment, medium was replaced with fresh one lacking phenol red and riboflavin (FluoroBrite, Thermo Fisher Scientific). Cells were imaged on a Nikon Ti inverted fluorescence microscope with a Nikon DS-Qi2 camera and a 20× plan apo objective (NA 0.75) using appropriate filter sets (CFP: 438/24 nm excitation (EX), 458 nm dichroic beam splitter (BS), 483/32 nm emission (EM); mVenus: 500/24 nm EX, 520 nm BS, 542/27 nm EM). We acquired images every 15 min for the duration of 24 h using Nikon Elements software. The microscope was surrounded by an enclosure to maintain constant temperature (37°C), CO_2_ concentration (5%), and humidity (OkoLab). The inhibitors were prepared in 500 μl media and added them, if not specified otherwise, 1 h before irradiation to achieve the final concentration in 2.5 ml media. We started imaging 30 min after irradiation. For experiments in the 24 well plate format, we prepared the inhibitors in 125 μl media and added them 1 h before irradiation to get the final concentration in a total volume of 1.125 ml media. Here, imaging was started directly after irradiation.

### Image and data analysis

Cells were tracked throughout the duration of the experiment using custom-written Matlab (MathWorks, Natick, MA) scripts based on code developed by the Alon lab [66] and the CellProfiler project [67]. In brief, we applied flat-field correction and background subtraction to raw images before segmenting individual nuclei from nuclear marker images using adaptive thresholding and seeded watershed algorithms. Segmented cells were then assigned to corresponding cells in following images using a greedy match algorithm. Only cells tracked from the first to last time point were considered. Cells were tracked in forward direction. Upon cell division, we followed the daughter cell closest to the last position of the mother and merged tracks from mothers and offspring. We quantified the nuclear fluorescence intensity of p53-Venus for each cell over time and analyzed the resulting single-cell trajectories. For further details on data preprocessing, see Strasen et al. 2018 [41] and S1 Text.

### Western blot analysis

We harvested cells at the indicated time points and lysed them in the presence of protease and phosphatase inhibitors. After extraction, proteins were quantified using Bradford assay (Carl Roth). Equal protein amounts were separated by electrophoreses on 4–12% Bis-Tris gradient gels (Thermo Fisher Scientific) and transferred to PVDF membranes (Thermo Fisher Scientific) by electroblotting. We blocked membranes with 5% nonfat dried milk (Carl Roth) or 5% bovine serum albumin (Carl Roth) and incubated them overnight with primary antibody. The next day, we washed the membranes, incubated them with secondary antibody coupled to peroxidase, and washed them again. Protein levels were detected using chemiluminescence (ECL Prime, GE Healthcare or WesternBright Quantum, Biozym).

We used antibodies against p53 (DO-1, sc-126), Mdm2 (SMP14, sc-965), Wip1 (H-300, sc-20712) from Santa Cruz Biotechnology (Dallas, TX), phospho-Chk2 (Thr-68), pSTAT3 (Tyr-705) from Cell Signaling (Danvers, MA) and glyceraldehyde-3-phosphate dehydrogenase (GAPDH) from Sigma-Aldrich.

### Reverse transcriptase quantitative PCR

We harvested cells at the indicated time points and extracted mRNA using High Pure RNA Isolation kits (Roche, Mannheim, Germany). Concentrations were measured with a photospectrometer (NanoDrop 2000, Thermo Fisher Scientific). We generated cDNA from 1μg RNA using M-MuLV reverse transcriptase (NEB, Ipswich, MA) and oligo-dT primers. Quantitative PCR was performed in triplicates using SYBR Green reagent (Roche) on a StepOnePlus PCR machine (Thermo Fisher Scientific). We used the following primers: β-actin forward (GGC ACC CAG CAC AAT GAA GAT CAA), β-actin reverse (TAG AAG CAT TTG CGG TGG ACG ATG), Wip1 forward (ATA AGC CAG AAC TTC CCA AGG), Wip1 reverse (TGG TCA ATA ACT GTG CTC CTT C), Mdm2 forward (GAT GAA AGC CTG GCT CTG TGT GT), Mdm2 reverse (TTC GAT GGC GTC CCT GTA GAT TCA), p21 forward (TGG ACC TGT CAC TGT CTT GT), p21 reverse (TCC TGT GGG CGG ATT AG), GADD45a forward (GCA ATA TGA CTT TGG AGG AAT TCT C), GADD45a reverse (TGA CTC AGG GCT TTG CTG), PUMA forward (CGA CCT CAA CGC ACA GTA CG), PUMA reverse (GGG TGC AGG CAC CTA ATT GG), PML forward (AGA CTC AGA TGC CGA AAA CTC), PML reverse (GGT CAG CAA GGT TCT CGT C) and NOXA forward (GGA GAT GCC GCC TGG GAA GAA G), NOXA reverse (TGC CGG AAG TTC AGT TTG TC).

### Immunofluorescence

We seeded cells on coverslips coated with poly-l-lysine (Sigma-Aldrich). The day of the experiment, cells were treated as indicated, fixed with 2% paraformaldehyde (Carl Roth) and permeabilized with 0.1% Triton X-100 (Carl Roth) in phosphate-buffered saline. We blocked with 10% goat serum (PAN-Biotech) and incubated with antibody against p65 (C-20, sc-372) from Santa Cruz Biotechnology (Dallas, TX). Cells were washed, incubated with secondary antibody coupled to Alexa Fluor 488 (Thermo Fisher Scientific) and washed again. Finally, cells were stained with Hoechst and embedded in Prolong Antifade (Thermo Fisher Scientific). We acquired images with a 20× Plan Apo objective (NA 0.75) using appropriate filter sets (mVenus: 500/24 nm EX, 520 nm BS, 542/27 nm EM, DAPI: 387/11 nm EX, 409 nm BS, 447/60 nm EM). Automated segmentation was performed in Matlab (The Mathworks Inc., Natick, MA) with algorithms from CellProfiler [67].

### Mathematical modeling

For simulations and parameter inference Matlab (R2017b, The Mathworks Inc., Natick, MA) and the open source toolbox Data2Dynamics [68,69] were used. Descriptions of model parameters as well as the inferred parameter values are given in S1 Table. For additional information and detailed description of methods, see S1 Text.

## Supporting information

S1 Text Supporting information on data sets and applied methods. In this supplement, additional information is given on time lapse microscopy data sets, quantification of features of dynamics, clustering of trajectories, subpopulation-based modelling, parameter inference, sensitivity analyses, as well as calculation of the weighted χ^2^ value.
(PDF)

S1 FigDefinition of features of p53 dynamics and reproducibility of results.a) Immunofluorescence was performed in A549 cells treated with TNFα, DMSO or IKK2i showing p65 translocation. b) Quantification of immunofluorescence data acquired in A549 cells treated with DMSO or IKK2i before irradiation with 10 Gy showing p65 translocation. c) Scheme of the defined features of p53 dynamics. Depicted are the timing of maxima (t^max^), timing of minima (t^min^), inter-peak-interval (IPI), dampening factor (DF), absolute values of maxima (F^max^), absolute values of minima, peak width, positive slope of peaks, negative slope of peaks and amplitude of peaks. The absolute values of maxima (F_n_^max^) are used to calculate the dampening factor (${DF}_{n}=\frac{F_{1}^{max}}{F_{n}^{max}})$ for peak *n*. d) The raw and smoothed trajectories of four random cells are depicted in blue and orange, respectively. The trajectories are smoothed by using a gaussian-weighted moving average. e) Distributions for the peak timing of the first five peaks are illustrated for raw and smoothed trajectories. f) A549 reporter cells were tracked and the p53 median nuclear fluorescence intensity was measured in cells treated with 10 Gy IR in combination with DMSO or IKK2i. Three independent experiments were performed, the cell numbers of each replicate are given in brackets. g) Quantification of selected features (timing maxima, timing minima, inter-peak-intervals and dampening factor) of the first four p53 pulses. The significance was tested by using the Wilcoxon rank sum test in combination with the Bonferroni-Holm method to correct for multiple testing, *p<0.05, **p<0.01, ***p<0.001.(PDF)

S2 Fig Validation and specificity of IKK2 inhibitors. a) Western blot analysis of p53 and GAPDH upon 10 Gy IR in A549 cells treated with DMSO or IKK2i. b) Using live-cell time-lapse microscopy, A549 reporter cells were tracked and the p53 median nuclear fluorescence intensity was measured upon 0 Gy or 10 Gy IR in cells treated with DMSO or IKK2i. c) A549 reporter cells were tracked and the p53 median nuclear fluorescence intensity was measured upon 10 Gy IR in cells treated with DMSO or different IKK2 inhibitors. d) The specificity of IKK2 inhibition was tested by comparing the effect of the IKK2 inhibitor TPCA-1 on features of p53 dynamics with the effects of two structurally independent IKK2 inhibitors (BMS and SC). For comparison, 17 criteria were defined which are derived from altered features, induced by application of TPCA-1. A green box indicates that an inhibitor induced the same specified effect as the TPCA-1 inhibitor. A red box represents a mismatch for the observed effect of the specified inhibitor. e) Western blot analysis of pSTAT3 and GAPDH in A549 cells treated with DMSO or the corresponding IKK2i prior to IL-6 addition. f) Using live-cell time-lapse microscopy, A549 reporter cells were tracked and the p53 median nuclear fluorescence intensity was measured upon 10 Gy IR in cells treated with DMSO, IKK2i or IL-6.
(PDF)

S3 Fig Sensitivity analysis for all 22 kinetic parameters, which are shared among the models of the model pool. Boxes represent the computed sensitivity coefficient for specified parameters, subpopulations and features for a single peak. The color of boxes depicts the value of the sensitivity coefficient. For the sensitivity analysis the first four peaks were evaluated.
(PDF)

S4 Fig Sensitivity analysis for single parameters for a perturbation strength of +30%. Changes in features that exceed a threshold of 1·10^−3^ are depicted in red if the change is positive and blue if the change is negative. Grey boxes indicate changes in features below 1·10^−4^. The corresponding parameter perturbation is considered to have no considerable effect. Sensitivity coefficients of peaks that are not consistently changed are depicted in white.
(PDF)

S5 Fig Sensitivity analysis for single parameters for a perturbation strength of -30%. Changes in features that exceed a threshold of 1·10^−3^ are depicted in red if the change is positive and blue if the change is negative. Grey boxes indicate changes in features below 1·10^−4^. The corresponding parameter perturbation is considered to have no considerable effect. Sensitivity coefficients of peaks that are not consistently changed are depicted in white.
(PDF)

S6 FigAssigning trajectories of the perturbation data to the ten subpopulations of the calibration data.a) Blue dots and lines represent the peak-based mean of trajectories from the calibration data. The red dots and lines depict the peak-based mean of trajectories from the perturbation data, assigned to the respective subpopulation. The number of trajectories which are assigned to a subpopulation and used for computing the peak-based mean, are stated by the cell numbers in the upper right corner of each subpopulation plot. The first number denotes the subpopulation-specific number of cells for the calibration data, the second number accounts for the perturbation data. The peak-based mean for the total population is shown in the lower right corner of the figure and was determined by calculating the weighted mean over all subpopulations. The weight is given by the number of cells assigned to a subpopulation. b) Evaluation of fulfilled criteria of the peak-based mean of the perturbation data. Green boxes indicate that a certain criterion, described on the left-hand side, is fulfilled by an individual subpopulation (*a* to *j*). Red boxes represent unmet criteria. c) The percentages denote the relative amounts of cells assigned to a subpopulation for the calibration data (IR+DMSO) and the perturbation data (IR+IKK2i). d) The grey boxes represent the ten subpopulations. To determine the impact of a subpopulation on the model fit, the sum of cells assigned to a subpopulation of both conditions (IR+DMSO and IR+IKK2i) is normalized to the total amount of cells in both conditions. Deviation *d* for a subpopulation is determined by the difference in percentages (*f*) between both conditions which is normalized to the maximal percentage of the two conditions: $d=\frac{{|f}_{IR}-f_{IKK2i}|}{max(\{f_{IR},f_{IKK2i}\})}*100\%$.(PDF)

S7 Fig Fitting parameter triplets to the perturbation data allows to reproduce the modulated p53 dynamics upon IKK2 inhibition. a) Simulation of the best fit of all tested parameter pairs. For a better visualization, the weighted mean over all subpopulations is shown for the simulation (red line) and the peak-based mean (black line with dots). b) Each dot represents a combination of parameter pairs (light red) or triplets (dark red) and the corresponding discrepancy between simulation and experimental data. c) The plots show simulations of three representative parameter combination fits, resulting in different fit qualities.
(PDF)

S8 Fig Simulations of the 30 best ranked parameter combination fits. The black line with dots represents the peak-based mean. The red line depicts the simulation of the specified parameter combination fit. For a more compact visualization, the peak-based mean and the simulation of individual subpopulations is represented by the weighted mean, which is determined by averaging over all subpopulations. The weight is derived from the number of cells assigned to a subpopulation.
(PDF)

S9 Fig Time-variant IKK2 inhibition used to validate the 30 best ranked parameter combinations. The experimental data (black dots) shows mean p53 dynamics upon IR and IKK2 inhibition at the specified time points. Simulations of four selected parameter combinations are represented by the colored lines, denoting the weighted mean of subpopulation dynamics. The index of each parameter combination derived from the corresponding summarized log_10_ χ^2^ value (Fig 5b) is given by the number in brackets.
(PDF)

S10 Fig Mechanisms of crosstalk in the p53 network. Western blot analysis of Wip1 and Mdm2 (a) as well as pChk2 (b) and GAPDH upon 10 Gy IR in A549 cells treated with DMSO or IKK2i. c) Summary of previously reported interactions between IKK2 and p53.
(PDF)

S1 Table Description and estimated values of parameters of the calibrated model pool. (PDF)
