# Supplementary material for: A systematic approach to decipher crosstalk in the p53 signaling pathway using single cell dynamics
Source: PLoS Comput Biol. 2020 Jun 26;16(6):e1007901. doi: 10.1371/journal.pcbi.1007901 (PMC7319280; doi:10.1371/journal.pcbi.1007901)
Supplement: S1 Text — In this supplement, additional information is given on time lapse microscopy data sets, quantification of features of dynamics, clustering of trajectories, subpopulation-based modelling, parameter inference, sensitivity analyses, as well as calculation of the weighted χ2 value. (PDF) [file pcbi.1007901.s001.pdf]

I. Single cell time lapse microscopy data sets

In this study, we used four single cell data sets comprising multiple experimental conditions. An overview of the data sets is shown in Table S1. The table includes experimental conditions, corresponding figures, number of trajectories and number of discarded trajectories due to filtering. For the reference data set and the inhibitor data set a filter for trajectories reaching a detection limit was applied (section II.B). For the calibration data set and perturbation data set a filter for outliers regarding peak number and peak timing was applied (III.A) as well as a filter for clusters with a low number of cells (III.D).

Table S1. Description of single cell data sets.

**label**	**conditions**	**description**	**Figures**	**number of discarded trajectories/overall trajectories (%)**
reference data	IR+DMSO<br>IR+TNFα<br>IR+IKK2i (TPCA-1)	impact of NF-κB activation status on p53 dynamics	1a-d	25/521 (5%)<br>5/611 (1%)<br>21/367 (6%)
inhibitor data	IR+DMSO<br>IR+IKK2i (BMS)<br>IR+DMSO<br>IR+IKK2i (SC)	testing consistency of changed features upon inhibition of IKK2 using different IKK2 inhibitors	S2c, S2d	4/178 (3%)<br>75/1011 (7%)<br>37/780 (5%)<br>24/1102 (2%)
time-variant IKK2 inhibition data	IR+DMSO<br>IR+IKK2i (t_{Inh}=1.5h, t_{Inh}=1.5h, t_{Inh}=2.5h, t_{Inh}=3h, t_{Inh}=5h, t_{Inh}=5h)	validation of model predictions	5, S9	-
calibration data<br>perturbation data	IR+DMSO<br>IR+IKK2i (TPCA-1)	parameter inference	3, S7, S6a, S8	93/374 (24.9%)<br>118/348 (33.9%)

II. Quantifying features of single cell dynamics

Single cell trajectories were processed and filtered in order to improve the quantification of features of dynamics.

A. Smoothing of single cell trajectories

In order to remove spikes in trajectories, a gaussian-weighted moving average with a window size of seven was applied to all trajectories, using the in-built function *smoothdata()* of Matlab. This way, identifying p53 peaks is less error-prone and evaluating dissimilarities between trajectories, which is important for clustering trajectories into subpopulations, is improved. As the window size of seven which corresponds to +/- 45 minutes is much smaller than the frequency of p53 oscillations (around 4.2h), high frequencies exhibited by spikes can be filtered out without affecting the pulses of p53 (Fig S1d). To assess the effect of the moving average on p53 dynamics, we determined the peak timing of the first five peaks for raw and smoothed trajectories (Fig S1e). While the moving average slightly increases the peak timing, the inter-peak interval and therefore the frequency is hardly changed (averaged inter-peak-interval: 4.15 hours (raw) compared to 4.18 hours).

B. Filter for trajectories reaching detection limits

Some data sets contain trajectories in which a fluorescent intensity threshold was reached. Such trajectories were excluded from further analysis, as it is not possible to detect peaks precisely. The trajectories were identified by checking for intensity values of a trajectory occurring multiple times and having identical numerical values. Such values were identified as intensity limit and affected trajectories were neglected.

C. Quantification of features and determining statistical significance

For quantification of features of dynamics we developed and used a custom-written Matlab script. It allows to detect minima and maxima of a trajectory by evaluating the sign of slopes of a trajectory (see also Fig S1c). In particular, the sign of a slope between two consecutive data points is determined by calculating the difference between absolute values of the two data points. After calculating the sign of slopes for all data points, extrema can be found by identifying a change in the sign of slopes. Based on the identified extrema, features of dynamics were calculated. The inter-peak-interval (IPI), dampening factor (DF) and slopes (s) of peaks are calculated as follows:

$$IPI_n = t_{n+1}^{max} - t_n^{max}$$

$$DF_n = \frac{F_1^{max}}{F_n^{max}}$$

$$s_n^+ = \frac{F_n^{max} - F_n^{min}}{t_n^{max} - t_n^{min}}$$

$$s_n^- = \frac{F_n^{max} - F_{n+1}^{min}}{t_n^{max} - t_{n+1}^{min}}$$

where t_n^{max} is the timing of the nth maximum, F_n^{max} denotes the absolute value of the nth maximum. The positive slope (s_n^+) is determined by the absolute values of the nth maximum and nth minimum (F_n^{min}) as well as the timing of these extrema ($t_n^{min} < t_n^{max}$). The amplitude of a maximum is defined as the difference between the absolute value of the maximum and the intersection of a line connecting the two surrounding minima and a vertical line going through the time point of the maximum (Fig S1c). The width of a peak is determined at the level of half the amplitude. In order to evaluate if an individual feature of dynamics is significantly changed, the Wilcoxon rank sum test was used [1,2]. For application of the rank sum test, the Matlab in-built function *ranksum()* was used. As multiple comparisons of features were performed, the calculated significance was corrected by applying the Bonferroni-Holm method [3]. For the correction, we used an open-source script for Matlab: Groppe, David (2010). Bonferroni-Holm Correction for Multiple Comparisons (https://de.mathworks.com/matlabcentral/fileexchange/ 28303-bonferroni-holm-correction-for-multiple-comparisons), Matlab Central File Exchange.

III. Clustering of single cell trajectories

For the subpopulation-based modeling approach, the single cell trajectories were clustered into subpopulations based on similarity of dynamics. After processing and filtering the trajectories, the similarity was determined and clustering was performed.

A. Processing and filtering of trajectories

In order to improve clustering, the trajectories of the calibration data set were smoothed (II.A), features of dynamics were quantified (II.C) and a filter was applied to detect outliers regarding peak number and peak timing. In particular, to promote small within-cluster variances while preventing high number of clusters, trajectories with strongly deviating numbers of peaks or strongly deviating timings of peaks compared to mean values of the population, were excluded from further analyses. Trajectories were excluded if:

$$n_i < \mu - 2\sigma$$

where n is the number of peaks of trajectory i, μ is the mean number of peaks of all trajectories with the standard deviation σ.

To exclude trajectories with deviating timing of peaks, the timing of maxima for each trajectory was analyzed:

$$-2\sigma + \mu < t_{i,n}^{max} < 2\sigma + \mu$$

where μ represents the mean timing of maxima (t_n^{max}) for trajectory i and peak n. The standard deviation of timing of maxima is represented by σ. The number of excluded cells for each data set is shown in Table S1.

B. Determining dissimilarities between single cell trajectories

Inspired by Strasen et al. [4], we applied dynamic time warping (DTW) [5] to determine the dissimilarity between trajectories. By employing DTW, two trajectories are aligned by nonlinear scaling which allows to compensate time shifts between two trajectories. This way, minor shifts in dynamics do not corrupt the calculated dissimilarities but promote the identification of similar patterns in dynamics. Without restricting DTW, alignment of two highly different trajectories can result in a strong adaption of trajectories. Such alignments are inconsistent with physiological differences in pulsatile dynamics of p53 which are assumed to be in a time frame of a few hours. Therefore, we used a Sakoe-Chiba band for DTW which restricts the alignment to a specified value and thereby ensures to identify similar patterns of dynamics in a certain time frame. A band width of five data points was chosen for the Sakoe-Chiba band, corresponding to a delay of 75 minutes. Using DTW, dissimilarities of all trajectories were determined in a pair-wise manner. The resulting dissimilarity matrix can then be used to cluster trajectories.

C. Clustering

For clustering, an agglomerative hierarchical clustering algorithm was used. It is a bottom-up approach starting with each provided data point (i.e. difference between two trajectories) as a cluster. We used Ward's method [6] which was shown to perform well in clustering single cell dynamics [4]. The Matlab in-built function *linkage()* was used to generate a hierarchical tree of the data. Subsequently, the hierarchical tree was passed to the function *cluster()*.

In order to identify the number of clusters in the calibration data set, we used the Calinski-Harabasz index [7]. By evaluating the between-cluster and within-cluster

variance the Calinski-Harabasz index allows to identify the optimal number of clusters. We determined the index for 1 to 40 clusters by using the Matlab in-built function *evalclusters().* The highest value for the index, indicating the optimal number of clusters, was found to be 23 in the calibration data set. We also tested the silhouette value [8] and the jump method [9] to identify the optimal number of clusters. However, calculating the silhouette value suggested 2 clusters which results in a high within-cluster variance and therefore a poor representation of single cell dynamics. The jump method suggested 38 clusters which would result in high computational costs for our modeling effort as the number of subpopulation-specific parameters is dependent on the number of clusters.

D. Filter for clusters with a low number of assigned cells

After clustering the trajectories into 23 different clusters, the number of trajectories which are assigned to a cluster can be very different. To reduce the number of subpopulation-specific parameters and therefore the computational costs, we excluded clusters with less than ten assigned trajectories from further analyses. For parameter inference the averaged dynamics of clusters were weighted based on the number of assigned cells (IV.F). Hence, the excluded clusters and trajectories would only have a minor effect on parameter inference.

E. Determining the peak-based mean

Upon clustering of trajectories, an appropriate measure had to be used to represent the clustered dynamics. Due to phase shifts in oscillations of p53, the mean or median is a poor representation of the clustered trajectories. Thus, we calculated a mean of dynamics which is not based on time points but on the peaks of single cell trajectories. In particular, we determined the maxima and minima of each trajectory of a cluster and calculated the mean of the timing of the extrema and the mean of absolute values of the extrema. Additionally, we identified time points between timings of maxima and preceding minima ($t_{0.5}^{-}$) and between timings of maxima and subsequent minima ($t_{0.5}^{+}$):

$$t_{0.5}^{-} = t_{\max} - 0.5(t_{max} - t_{min}^{-})$$

$$t_{0.5}^{+} = t_{\max} + 0.5(t_{min}^{+} - t_{max})$$

where $t_{0.5}^{-}$ represents the time point which is in between the timing of a maximum (t_{max}) and a preceding minimum (t_{min}^{-}). In contrast, $t_{0.5}^{+}$ denotes the timing between a maximum and a subsequent minimum (t_{min}^{+}). After identifying the timings between

maxima and minima, the corresponding absolute values of the trajectory at these time points were identified. Again, the mean of timings and absolute values was determined. Finally, all calculated mean data points were combined and resulted in a trajectory, representing the dynamics of single cell trajectories assigned to a cluster and denoted as peak-based mean.

IV. Subpopulation-based modelling

A. Model describing p53 signaling

The model which was used for subpopulation modeling is based on the model of Batchelor et al. [10,11]. It is a delay differential equation (DDE) model, in which transcription and translation of p53 target genes are described by delay differential equations. As we were interested in a detailed description of processes affecting the regulators of p53 and parameter inference is computationally more efficient for an ODE model, we replaced the DDEs by ODEs. In particular, we included variables for Mdm2 mRNA and Wip1 mRNA as well as transcription and translation processes for Mdm2 mRNA and Wip1 mRNA. In addition, we also introduced a basal transcription of the Wip1 gene to account for a basal amount of Wip1 in steady state.

B. Model parameters

The units of the parameters are given in arbitrary concentration *c* and hours *h*. The boundaries of kinetic parameters were set to -10 and 4 on a log10 scale if not stated otherwise. The Hill coefficients *ns* and *nw* were not fitted to the data but fixed to the values which were used by Batchelor et al. [10,11]. The boundaries of parameters describing the degradation of Mdm2 mRNA, Wip1 mRNA, Mdm2 and Wip1 were set according to literature data. Porter et al. [12] and Hafner et al. [13] reported for MCF7 cells a degradation rate for Mdm2 mRNA of 0.29 h^{-1} and 0.5 h^{-1}, respectively. Schwanhausser et al. [14] quantified the half-life of Mdm2 mRNA in mouse embryonic fibroblasts and reported a half-life of 3.5 h which corresponds to a degradation rate of 0.2 h^{-1}. Consequently, the boundaries for parameter α_{mt} were set to 0.1 h^{-1} and 1 h^{-1}.

The degradation rate of Wip1 mRNA, which corresponds to parameter α_{wt}, was determined by Porter et al. [12] in MCF7 cells to be 0.65 h^{-1}. Again, the boundaries were set to 0.1 h^{-1} and 1 h^{-1}.

Chang et al. [15] and Finlay et al. [16] quantified 0.5 h as half-life for Mdm2 in HeLa cells and rat embryonic cells, respectively. The corresponding degradation rate is 1.4 h^{-1} and therefore the boundaries for parameter α_m were set to 1 h^{-1} and 5 h^{-1}.

The half-life of Wip1 was reported by Choi et al. [17] and Kleiblova et al. [18] to be in the range of 1 h and 2 h in HCT116 and U2OS cells, corresponding to a degradation rate of 0.69 h^{-1} and 0.35 h^{-1} respectively. Thus, the boundaries for parameter α_w were set to 0.1 h^{-1} and 1 h^{-1}. The estimated parameter values and parameter boundaries from the calibrated model pool are shown in Fig S11.

C. Generating pool of ODE models

A pool of ODE models was generated by defining subpopulation-specific parameters for processes assumed to be susceptible to noise. The models can have different parameter values for these processes and therefore allow to simulate different p53 dynamics. As it could be shown that cell-to-cell variability can be explained by differences in protein and mRNA levels [19,20], we defined the parameters of RNA and protein synthesis rates as subpopulation-specific. The number of models in the model pool depend on the number of identified subpopulations in the data set. As we identified ten subpopulations in the calibration data set, we generated ten models which differ in the values of the subpopulation-specific parameters (Fig 2a,b). These parameters were implemented as a fold change which is multiplied with a parameter that is shared among all models. This way, the magnitude of variations across subpopulation-specific parameter values can be controlled and limited to a physiological range. The boundaries of fold change parameters were set to $10^{-1.5}$ and $10^{1.5}$. Subpopulation-specific fold change parameters are indicated by the suffix *fc* and a character from *a* to *j* representing the subpopulation.

The following ODE system describes one model of the model pool which is specific for subpopulation *a*. The remaining models of the model pool contain the same ODEs but differ in the subpopulation-specific parameters.

In order to simulate inhibition of IKK2 at different time points and thereby reflect the experimental setting of time-variant IKK2 inhibition, the inhibition was simulated by implementing a sigmoidal increasing effect of the inhibitor.

D. Ordinary differential equations

$$\frac{d(p53)}{dt} = v_1 + v_2 - v_3 - v_4 - v_5$$

$$\frac{d(p53a)}{dt} = -v_2 + v_5 - v_6$$

$$\frac{d(Mdm2_{mRNA})}{dt} = v_7 + v_8 - v_9$$

$$\frac{d(Mdm2)}{dt} = v_{10} - v_{11} - v_{12}$$

$$\frac{d(Wip1_{mRNA})}{dt} = v_{13} + v_{14} - v_{15}$$

$$\frac{d(Wip1)}{dt} = v_{16} - v_{17}$$

$$\frac{d(ATMp)}{dt} = v_{18} - v_{19} - v_{20}$$

E. Reaction rates for model fitted to calibration data

$$v_1 = \beta_p \cdot \beta_{p\_}fca$$

$$v_2 = \alpha_{wpa} \cdot Wip1 \cdot p53a$$

$$v_3 = \alpha_{pi} \cdot p53$$

$$v_4 = \alpha_{mpi} \cdot Mdm2 \cdot p53$$

$$v_5 = \beta_{sp} \cdot p53 \cdot \frac{ATMp^{ns}}{ATMp^{ns} + T_s^{ns}}$$

$$v_6 = \alpha_{mpa} \cdot Mdm2 \cdot p53a$$

$$v_7 = \beta_{pamt} \cdot p53a$$

$$v_8 = \beta_{mt} \cdot \beta_{mt\_}fca$$

$$v_9 = \alpha_{mt} \cdot Mdm2_{mRNA}$$

$$v_{10} = \beta_{mtm} \cdot \beta_{mtm\_}fca \cdot Mdm2_{mRNA}$$

$$v_{11} = \alpha_{sm} \cdot Mdm2 \cdot ATMp$$

$$v_{12} = \alpha_m \cdot Mdm2$$

$$v_{13} = \beta_{pawt} \cdot p53a$$

$$v_{14} = \beta_{wt} \cdot \beta_{wt\_}fca$$

$$v_{15} = \alpha_{wt} \cdot Wip1_{mRNA}$$

$$v_{16} = \beta_{wtw} \cdot \beta_{wtw\_}fca \cdot Wip1_{mRNA}$$

$$v_{17} = \alpha_w \cdot Wip1$$

$$v_{18} = \beta_s \cdot \beta_{s\_}fca$$

$$v_{19} = \alpha_{ws} \cdot ATMp \cdot \frac{Wip1^{nw}}{Wip1^{nw} + T_w^{nw}}$$

$$v_{20} = \alpha_s \cdot ATMp$$

F. Fit of model pool to subpopulation data

For parameter inference, the open source toolbox Data2Dynamics (D2D) for Matlab (R2017b, The Mathworks Inc., Natick, MA) was used [21,22]. The optimization is based on the deterministic *lsqnonlin* algorithm which is implemented in Matlab. Multi start optimization was performed by sampling start values of parameters using latin hypercube sampling [23,24]. By defining the subpopulation-specific parameters in the toolbox as condition-specific, it is possible to fit simultaneously shared and specific parameters to the experimental data. In order to include the information on the number of assigned cells to a cluster, we weighted the peak-based mean for each subpopulation accordingly. This was done by adapting the number of data points for each peak-based mean in accordance to the number of assigned cells.

G. Using L1 regularization to identify parameters with a major impact on heterogeneity

In the model, six processes were defined as subpopulation-specific. Defining for each subpopulation and each subpopulation-specific process a fold change parameter allowed to reproduce the heterogeneous dynamics of the different clusters. However, it is not clear if all of the subpopulation-specific fold change parameters are required to reproduce the data. In order to identify crucial subpopulation-specific parameters and processes to reproduce the heterogeneous dynamics, we applied L1 regularization [25]. Steiert et al. implemented this method in the D2D toolbox and successfully used this approach to identify cell type-specific parameters.

By including a penalty term for the parameter value of fitted parameters in the objective function of the optimization, regularization allows to detect parameters with a low or no impact on fit quality. Steiert et al. implemented the penalty term by introducing a logarithmic ratio of parameter values for each cell type-specific reaction. Consequently, by minimizing the objective function and thus the penalty term during optimization, the value of a logarithmic ratio is minimized. An optimal solution for a logarithmic ratio of zero indicates that the according reaction is not cell type-specific. The contribution of the penalty term to the objective function is regulated by a weight (λ) in order to control the balance between number of neglected parameters and goodness of fit.

To identify the optimal value for λ, Steiert et al. suggested to perform a likelihood ratio test. We tested 31 values for λ ranging from 10^{-20} to 10^{6}, and found an optimal value of $10^{2.7}$ for the weight λ.

For the optimal value of λ, 20 out of the 60 subpopulation-specific parameters were identified as unspecific. For basal transcription of the Wip1 gene (β_{wt}), all subpopulation-specific fold change parameters were estimated to be zero, indicating that this process is unspecific and thus not driving heterogeneity (Fig 3b).

Performing a profile likelihood estimation for the 40 identified subpopulation-specific fold change parameters revealed that all fold change parameters corresponding to activation of ATM (β_s) and translation of Wip1 mRNA (β_{wtw}) are structurally non-identifiable. Consequently, one parameter of each process can be fixed to zero without changing the overall goodness of fit. We selected β_s_fcb and β_{wtw}_fcb to be fixed to zero. The profile likelihood was again performed and showed that fixing the two parameters rendered all remaining subpopulation-specific parameters of activation of ATM and translation of Wip1 mRNA identifiable.

Taken together, L1 regularization in combination with profile likelihood analysis revealed that 22 out of the 60 defined subpopulation-specific fold change parameters are not crucial to reproduce the data and are therefore not driving the simulated heterogeneity (Fig 3b).

V. Sensitivity analysis

In order to assess the effect of a parameter on a specified feature of dynamics, e.g. timing of peak maxima, the model parameters were perturbed by +1% (or +30%, -30%) and the relative change in the feature for each peak was quantified. This way, a sensitivity coefficient (sc) can be computed, quantifying the effect of the analyzed parameter on the feature.

$$sc_i = \frac{\frac{\Delta f_i}{f_i}}{\frac{\Delta p}{p}}$$

where *p* represents the analyzed parameter and f is the quantified feature for peak *i*. To condense the sensitivity coefficients of multiple peaks, two thresholds were defined. One threshold was used to determine if a sensitivity coefficient is sensitive, i.e. the applied parameter perturbation has a considerable impact on the respective feature:

$$|sc_i| \geq 1 \cdot 10^{-3}$$

If the sensitivity coefficients of all peaks of a feature cross this threshold, the corresponding subpopulation is considered sensitive for the analyzed feature and parameter. In contrast, if the absolute value of sensitivity coefficients of all peaks are below the second threshold, the analyzed parameter perturbation is considered ineffective in changing a feature:

$$|sc_i| \leq 1 \cdot 10^{-4}$$

For the sensitivity analysis of parameter pairs, both parameters of a combination were perturbed by +1%, or one parameter was perturbed by +1% and the second one by -1% to account for opposing effects of IKK2 on two processes.

VI. Parameter inference of parameter pairs and parameter triplets to reproduce the perturbation data

After calibrating the subpopulation-based model pool, it can be used to evaluate parameters regarding their capability to reproduce the IKK2 inhibitor-induced effects on p53 dynamics. In order to analyze parameter pairs and parameter triplets in a quantitative manner, parameter inference was used. In particular, parameter combinations were fitted to the perturbation data while remaining parameters were fixed to their calibrated values. The resulting fit quality, assigned to each parameter combination, allowed to rank the combinations based on their capability to reflect the IKK2i-induced alterations in p53 dynamics. However, before the parameters can be fitted, the perturbation data had to be processed and single cell trajectories had to be assigned to clusters of the calibration data.

A. Processing of perturbation data

The perturbation data set in which cells were treated with IR and the IKK2 inhibitor were processed like the calibration data set. The trajectories were smoothed and filter for outliers regarding peak number and peak timing were applied (II.A and III.A).

B. Allocating trajectories of perturbation data to clusters of the calibration data

The calibration data was clustered into smaller subpopulations to preserve characteristics of single cell dynamics. The same was desired for the perturbation data. To track the effect of the inhibitor on the level of the subpopulations, the trajectories of the perturbation data were assigned to clusters of the calibration data. In particular, we quantified features of p53 dynamics of the calibration data and compared it to the

quantified features of the perturbation data. Based on the changes in features, we defined nine criteria (Table S2). By comparing features of single trajectories of the perturbation data to features of the peak-based mean of each cluster of the calibration data, the clusters can be ranked for each trajectory based on the defined criteria. The first eight criteria were used for the assignment to ensure that the observed effect of IKK2 inhibition on p53 dynamics are still represented on the subpopulation level. As multiple clusters can fulfill the first eight criteria for a trajectory, a ninth criterion was defined to unambiguously assign a trajectory to a cluster. The ninth criterion captures the difference between absolute values of the first maxima of p53 peaks between calibration and perturbation data. In particular, IKK2 inhibition did not change the absolute value of the first maximum significantly. Consequently, calculating the difference between the absolute value of the first maximum of a trajectory and the peak-based mean of each cluster allows to rank the clusters according to the quantified difference. The cluster exhibiting the smallest difference is assumed to be the most appropriate cluster for the analyzed trajectory based on the ninth criterion.

Assigning trajectories to clusters solely on basis of the ninth criterion, i.e. only the best ranked cluster is considered, would result in an unambiguous assignment but the respective trajectories would not necessarily reproduce the experimentally observed effects, defined by the first eight criteria. Thus, more than the best ranked cluster has to be considered. Moreover, applying all of the first eight criteria results in trajectories that cannot be assigned to a cluster due to unmet criteria and are therefore neglected for the assignment. Thus, it is important to choose an appropriate threshold for the number of fulfilled criteria to ensure that the IKK2-induced effect on dynamics is reflected while the number of neglected cells is small. To determine a threshold for i) the first eight criteria that have to be fulfilled and ii) a number of ranks, derived from the ninth criterion, that have to be considered we analyzed the effect of first eight criteria and the ninth criterion on the composition of assigned trajectories to clusters. This was done by calculating the adjusted rand index [26]. It is based on the rand index [27], a measure to compare compositions of two clustering realizations. For calculation of the adjusted rand index, we used an open-source script for Matlab: McComb, Chris (2015). Compute the adjusted rand index using compAdjRandIndex(), (https://de.mathworks.com/matlabcentral/fileexchange/49908-adjusted-rand-index), Matlab.

To compare all possible combinations of thresholds regarding their impact on the assignment of trajectories to a cluster, we defined one particular assignment as reference and compared all other assignments to this reference. The reference assignment is obtained by setting the threshold for the ranking to one (only best ranked cluster is used) and the threshold for the number of criteria to eight (each of the first eight criteria has to be fulfilled). By calculating the adjusted rand index between the tested thresholds and the reference realization we analyzed all possible thresholds. An adjusted rand index of one indicates a similar composition between two clustering realizations. The best setting resulting in a high number of the adjusted rand index and a low number of neglected cells was found by taking the first and second ranked cluster for a trajectory into account and setting the number of criteria that have to be fulfilled for the first eight criteria to four. The corresponding adjusted rand index was 0.64 while 23% of trajectories were neglected. With these settings it was possible to assign 77% of the trajectories of the perturbation data to the clusters of the calibration data. To test if the IKK2i-mediated changes in features are reflected by the determined peak-based mean, we compared the features of the peak-based mean of the calibration and perturbation data. Only subpopulation *b* failed to reflect the changes in features upon IKK2 inhibition (Fig S6b). This is due to a low number of assigned cells resulting in a higher variance in the quantified features. However, for fitting the model pool to the data, the peak-based mean of each subpopulation is weighted based on the number of assigned cells. Thus, subpopulation *b* has only a small impact on parameter inference compared to the other subpopulations.

Table S2. Criteria for trajectory assignment.

criteria	description	valid for
1	timing of maxima increased	all peaks
2	inter-peak-interval increased	all peaks
3	dampening factor increased	all peaks
4	absolute value of maxima decreased	peak 2 to 4
5	absolute value of minima decreased	peak 2 to 4
6	width increased	peak 1 and 2
7	positive slope decreased	all peaks
8	negative slope decreased	peak 2 to 4
9	absolute value of maxima unchanged (minimal difference)	peak 1

C. Parameter inference

After assigning trajectories of the perturbation data to the clusters of the calibration data, the calibrated model pool can be fitted to the peak-based mean of the assigned trajectories. The peak-based mean of subpopulations was weighted by taking the number of cells into account that were assigned to a subpopulation. This was done the same way as described for the peak-based mean of subpopulations of the calibration data (IV.F). Parameter inference was performed for all 231 possible combinations of 22 parameters pairs, as well as 1540 parameter triplets. In order to control the magnitude of inferred parameter values, a fold change parameter was introduced and multiplied with the parameter of interest. By fitting the fold change parameter and fixing all remaining parameters to their calibrated value, the fold change was inferred and restricted to be in a range between 10^{-5} and 10^{5}.

VII. Weighted χ^2 value

In order to quantify the difference between the validation data set of time-variant IKK2 inhibition and simulations of the model pool, the weighted χ^2 value was calculated. The χ^2 value was determined for each data set *i* and time point *j*:

$$\chi^2(t_j, p) = \frac{\left(\tilde{y}_{ij} - y_i(t_j, p)\right)^2}{\sigma_i^2(t_j)}$$

where $\tilde{y}$ defines the experimental data. The simulation data is denoted by *y* for parameters *p*. Note, the standard deviation *σ* is calculated for each measurement and time point t_j. For a better comparison of data sets, the χ^2 value of each data set was normalized to the smallest value of all time points *t* of this data set. The χ^2 value of a data set is given by the normalized sum of χ^2 values for all time points:

$$\bar{\chi}_i^2(p) = \sum_{j=1}^{n} \frac{\chi_{ij}^2(t_j, p)}{\min \chi_{ij}^2(t_j, p)}$$

The χ^2 values of two data sets with similar time points of IKK2 inhibition (1.5h/1.5h, 2.5h/3h and 5h/5h) were summarized by adding their normalized χ^2 values ($\bar{\chi}_i^2(p)$).

[1] F. Wilcoxon, Individual Comparisons by Ranking Methods, Biometrics Bull. 1 (1945) 80. doi:10.2307/3001968.

[2] H.B. Mann, D.R. Whitney, On a Test of Whether one of Two Random Variables is Stochastically Larger than the Other, Ann. Math. Stat. 18 (1947) 50–60. doi:10.1214/aoms/1177730491.

[3] Sture Holm, A Simple Sequentially Rejective Multiple Test Procedure, Scand. J. Stat. (1979). doi:10.2307/4615733.

[4] J. Strasen, U. Sarma, M. Jentsch, S. Bohn, C. Sheng, D. Horbelt, P. Knaus, S. Legewie, A. Loewer, Cell- specific responses to the cytokine TGFβ are determined by variability in protein levels, Mol. Syst. Biol. 14 (2018) e7733. doi:10.15252/msb.20177733.

[5] H. Sakoe, S. Chiba, Dynamic programming algorithm optimization for spoken word recognition, IEEE Trans. Acoust. Speech Signal Process. (1978). doi:10.1109/TASSP.1978.1163055.

[6] J.H. Ward, Hierarchical Grouping to Optimize an Objective Function, J. Am. Stat. Assoc. 58 (1963) 236–244. doi:10.1080/01621459.1963.10500845.

[7] T. Calinski, J. Harabasz, A dendrite method for cluster analysis, Commun. Stat. - Theory Methods. 3 (1974) 1–27. doi:10.1080/03610927408827101.

[8] P.J. Rousseeuw, Silhouettes: A graphical aid to the interpretation and validation of cluster analysis, J. Comput. Appl. Math. 20 (1987) 53–65. doi:10.1016/0377-0427(87)90125-7.

[9] C.A. Sugar, G.M. James, Finding the Number of Clusters in a Dataset, J. Am. Stat. Assoc. 98 (2003) 750–763. doi:10.1198/016214503000000666.

[10] E. Batchelor, C.S. Mock, I. Bhan, A. Loewer, G. Lahav, Recurrent initiation: a mechanism for triggering p53 pulses in response to DNA damage., Mol. Cell. 30 (2008) 277–89. doi:10.1016/j.molcel.2008.03.016.

[11] E. Batchelor, A. Loewer, C. Mock, G. Lahav, Stimulus-dependent dynamics of p53 in single cells., Mol. Syst. Biol. 7 (2011) 488. doi:10.1038/msb.2011.20.

[12] J.R. Porter, B.E. Fisher, E. Batchelor, p53 Pulses Diversify Target Gene Expression Dynamics in an mRNA Half-Life-Dependent Manner and Delineate Co-regulated Target Gene Subnetworks, Cell Syst. 2 (2016) 272–282. doi:10.1016/j.cels.2016.03.006.

[13] A. Hafner, J. Stewart-Ornstein, J.E. Purvis, W.C. Forrester, M.L. Bulyk, G. Lahav, p53 pulses lead to distinct patterns of gene expression albeit similar DNA-binding dynamics, Nat. Struct. Mol. Biol. 24 (2017) 840–847. doi:10.1038/nsmb.3452.

[14] B. Schwanhäusser, D. Busse, N. Li, G. Dittmar, J. Schuchhardt, J. Wolf, W. Chen, M. Selbach, Global quantification of mammalian gene expression control., Nature. 473 (2011) 337–42. doi:10.1038/nature10098.

[15] Y.C. Chang, Y.S. Lee, T. Tejima, K. Tanaka, S. Omura, N.H. Heintz, Y. Mitsui, J. Magae, Mdm2 and Bax, Downstream Mediators of the P53 Response, Are Degraded By the Ubiquitin-Proteasome Pathway., Cell Growth Differ. 9 (1998) 79–84.

[16] C.A. Finlay, The mdm-2 oncogene can overcome wild-type p53 suppression of transformed cell growth., Mol. Cell. Biol. 13 (1993) 301–6. http://www.ncbi.nlm.nih.gov/pubmed/8417333.

[17] D.W. Choi, W. Na, M.H. Kabir, E. Yi, S. Kwon, J. Yeom, J.W. Ahn, H.H. Choi, Y. Lee, K.W. Seo, M.K. Shin, S.H. Park, H.Y. Yoo, K. ichi Isono, H. Koseki, S.T. Kim, C. Lee, Y.K. Kwon, C.Y. Choi, WIP1, a Homeostatic Regulator of the DNA Damage Response, Is Targeted by HIPK2 for Phosphorylation and Degradation, Mol. Cell. 51 (2013) 374–385. doi:10.1016/j.molcel.2013.06.010.

[18] P. Kleiblova, I.A. Shaltiel, J. Benada, J. Ševčík, S. Pecháčková, P. Pohlreich, E.E. Voest, P. Dundr, J. Bartek, Z. Kleibl, R.H. Medema, L. Macurek, Gain-of-function mutations of PPM1D/Wip1 impair the p53-dependent G1 checkpoint, J. Cell Biol. 201 (2013) 511–521. doi:10.1083/jcb.201210031.

[19] S.L. Spencer, S. Gaudet, J.G. Albeck, J.M. Burke, P.K. Sorger, Non-genetic origins of cell-to-cell variability in TRAIL-induced apoptosis., Nature. 459 (2009) 428–32. doi:10.1038/nature08012.

[20] A. Loewer, G. Lahav, We are all individuals: Causes and consequences of non-genetic heterogeneity in mammalian cells, Curr. Opin. Genet. Dev. 21 (2011) 753–758. doi:10.1016/j.gde.2011.09.010.

[21] A. Raue, M. Schilling, J. Bachmann, A. Matteson, M. Schelke, D. Kaschek, S. Hug, C. Kreutz, B.D. Harms, F.J. Theis, U. Klingmüller, J. Timmer, Lessons learned from quantitative dynamical modeling in systems biology., PLoS One. 8 (2013) e74335. doi:10.1371/journal.pone.0074335.

[22] A. Raue, B. Steiert, M. Schelker, C. Kreutz, T. Maiwald, H. Hass, J. Vanlier, C. Tönsing, L. Adlung, R. Engesser, W. Mader, T. Heinemann, J. Hasenauer, M. Schilling, T. Höfer, E. Klipp, F. Theis, U. Klingmüller, B. Schöberl, J. Timmer, Data2Dynamics: a modeling environment tailored to parameter estimation in dynamical systems., Bioinformatics. (2015) 3–5. doi:10.1093/bioinformatics/btv405.

[23] M.D. McKay, R.J. Beckman, W.J. Conover, A Comparison of Three Methods for Selecting Values of Input Variables in the Analysis of Output from a Computer Code, Technometrics. 21 (1979) 239. doi:10.2307/1268522.

[24] A.B. Owen, A central limit theorem for Latin Hypercube Sampling, J. R. Stat. Soc. Ser. B (Statistical Methodol. (1992). doi:10.2307/2346140.

[25] B. Steiert, J. Timmer, C. Kreutz, L1 regularization facilitates detection of cell type-specific parameters in dynamical systems, Bioinformatics. 32 (2016) i718–i726. doi:10.1093/bioinformatics/btw461.

[26] L. Hubert, P. Arabie, Comparing partitions, J. Classif. 2 (1985) 193–218. doi:10.1007/BF01908075.

[27] W.M. Rand, Objective Criteria for the Evaluation of Clustering Methods, J. Am. Stat. Assoc. 66 (1971) 846. doi:10.2307/2284239.
