# Supplementary figures and images for: A systematic approach to decipher crosstalk in the p53 signaling pathway using single cell dynamics
Source: PLoS Comput Biol. 2020 Jun 26;16(6):e1007901. doi: 10.1371/journal.pcbi.1007901 (PMC7319280; doi:10.1371/journal.pcbi.1007901)

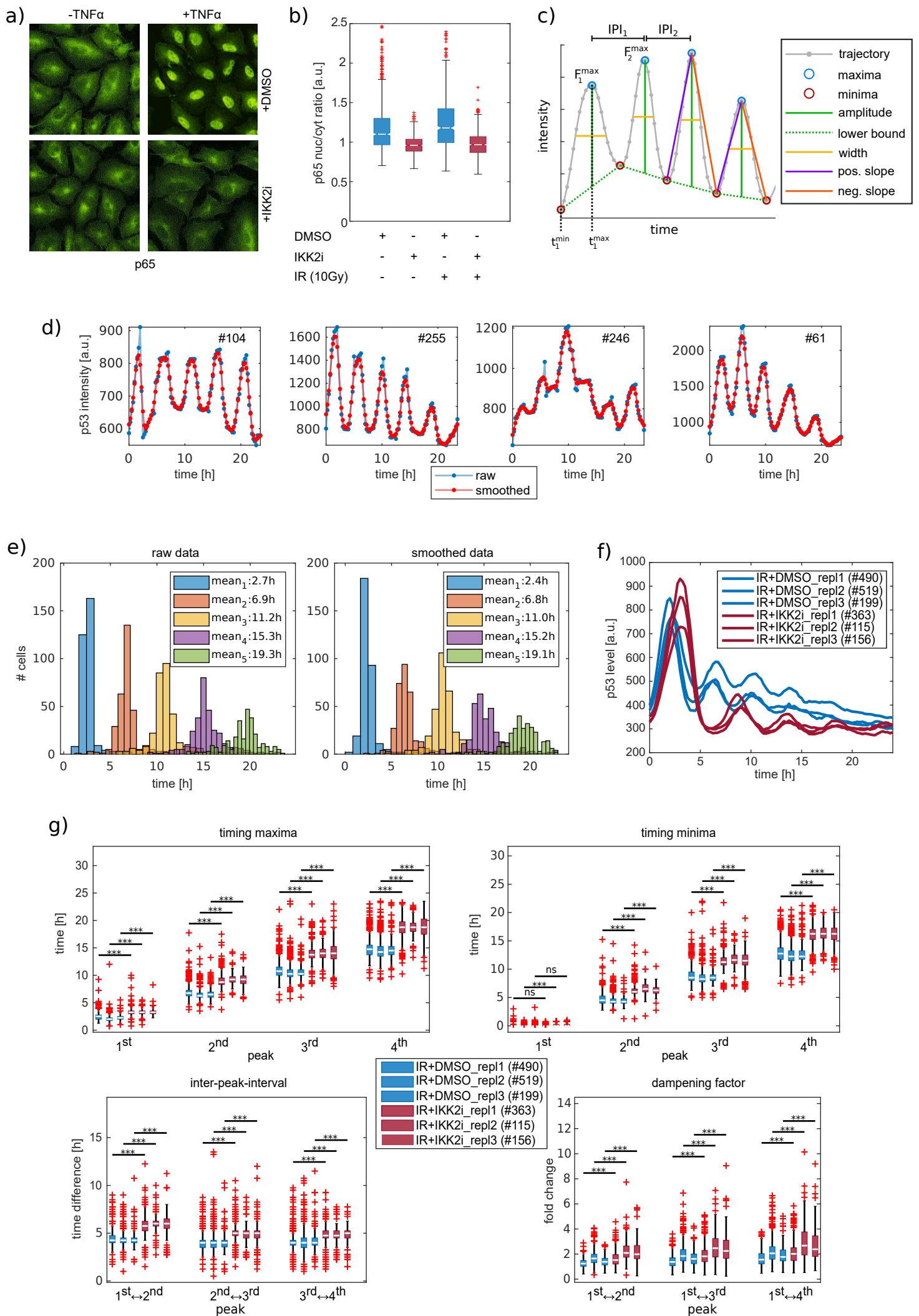

Supplement: S1 Fig — a) Immunofluorescence was performed in A549 cells treated with TNFα, DMSO or IKK2i showing p65 translocation. b) Quantification of immunofluorescence data acquired in A549 cells treated with DMSO or IKK2i before irradiation with 10 Gy showing p65 translocation. c) Scheme of the defined features of p53 dynamics. Depicted are the timing of maxima (tmax), timing of minima (tmin), inter-peak-interval (IPI), dampening factor (DF), absolute values of maxima (Fmax), absolute values of minima, peak width, positive slope of peaks, negative slope of peaks and amplitude of peaks. The absolute values of maxima (Fnmax) are used to calculate the dampening factor (DFn=F1maxFnmax) for peak n. d) The raw and smoothed trajectories of four random cells are depicted in blue and orange, respectively. The trajectories are smoothed by using a gaussian-weighted moving average. e) Distributions for the peak timing of the first five peaks are illustrated for raw and smoothed trajectories. f) A549 reporter cells were tracked and the p53 median nuclear fluorescence intensity was measured in cells treated with 10 Gy IR in combination with DMSO or IKK2i. Three independent experiments were performed, the cell numbers of each replicate are given in brackets. g) Quantification of selected features (timing maxima, timing minima, inter-peak-intervals and dampening factor) of the first four p53 pulses. The significance was tested by using the Wilcoxon rank sum test in combination with the Bonferroni-Holm method to correct for multiple testing, *p<0.05, **p<0.01, ***p<0.001. (PDF) [file pcbi.1007901.s002.pdf]

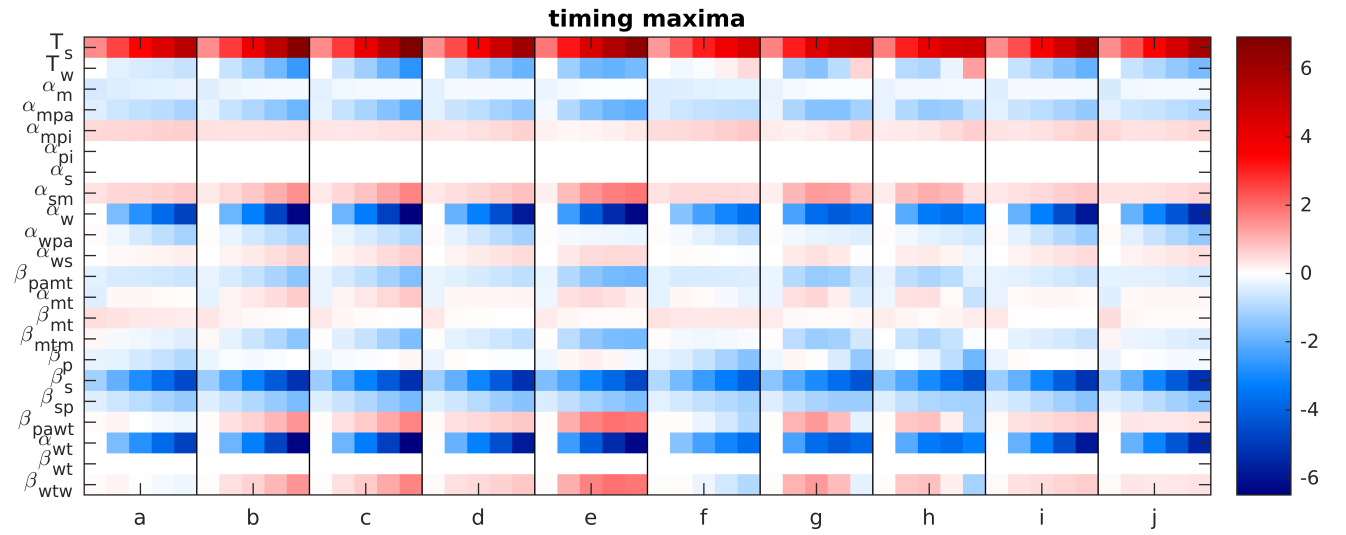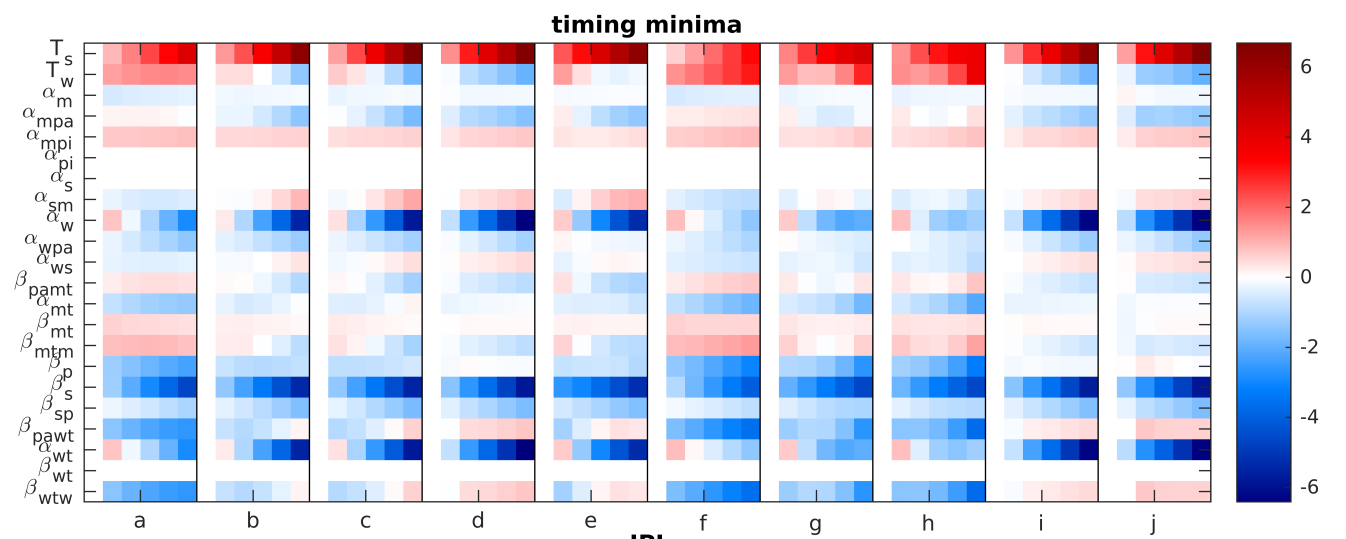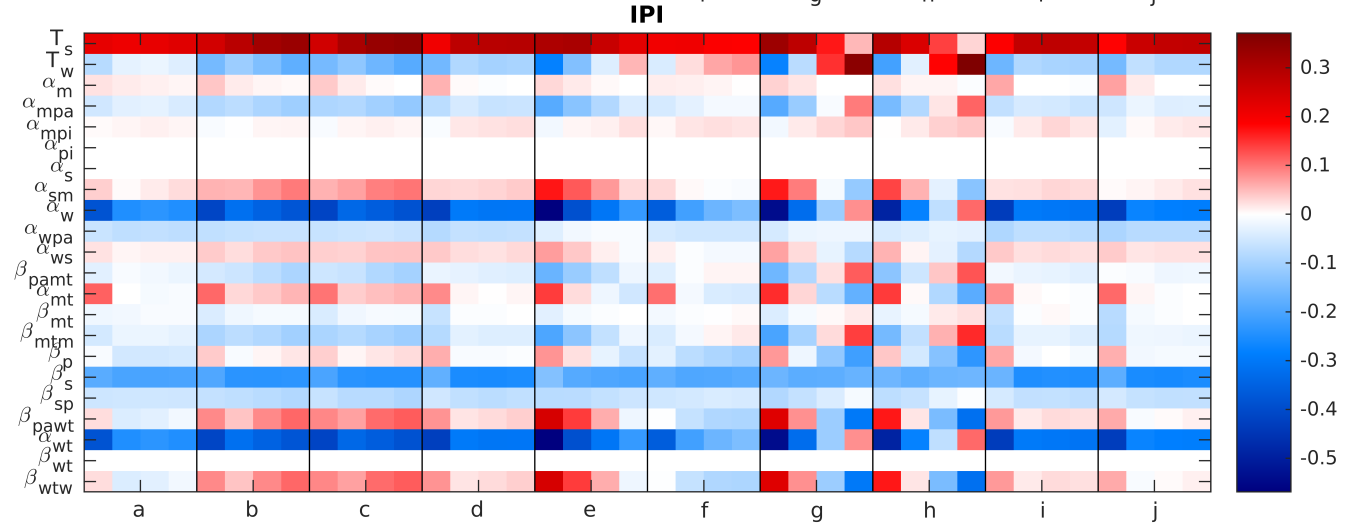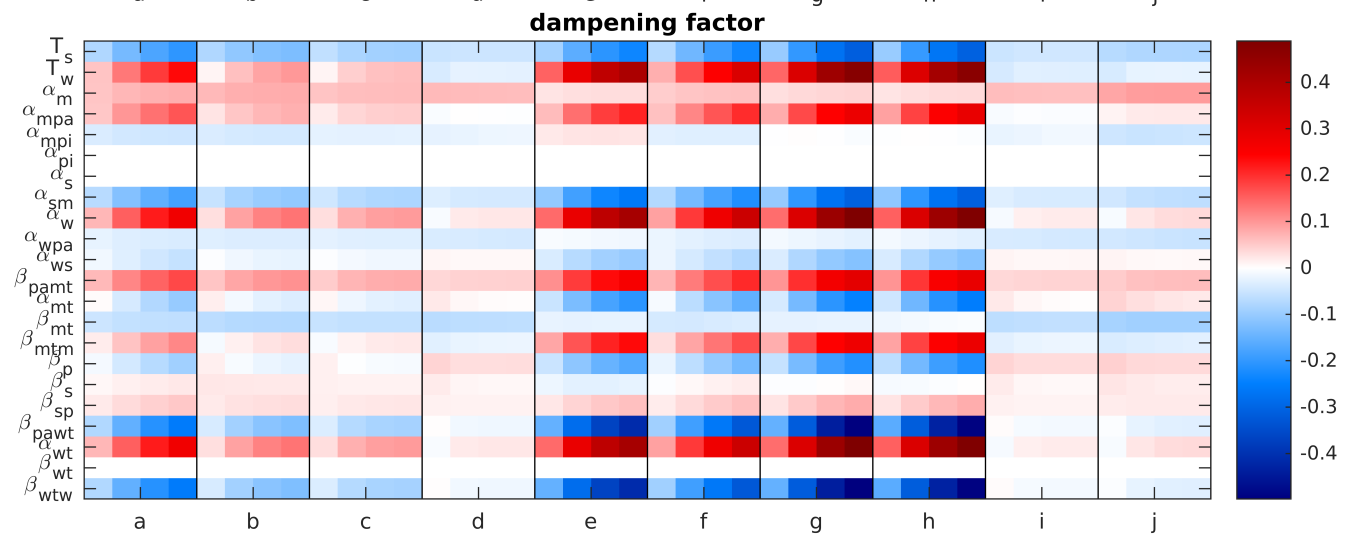

Supplement: S3 Fig — Boxes represent the computed sensitivity coefficient for specified parameters, subpopulations and features for a single peak. The color of boxes depicts the value of the sensitivity coefficient. For the sensitivity analysis the first four peaks were evaluated. (PDF) [file pcbi.1007901.s004.pdf]

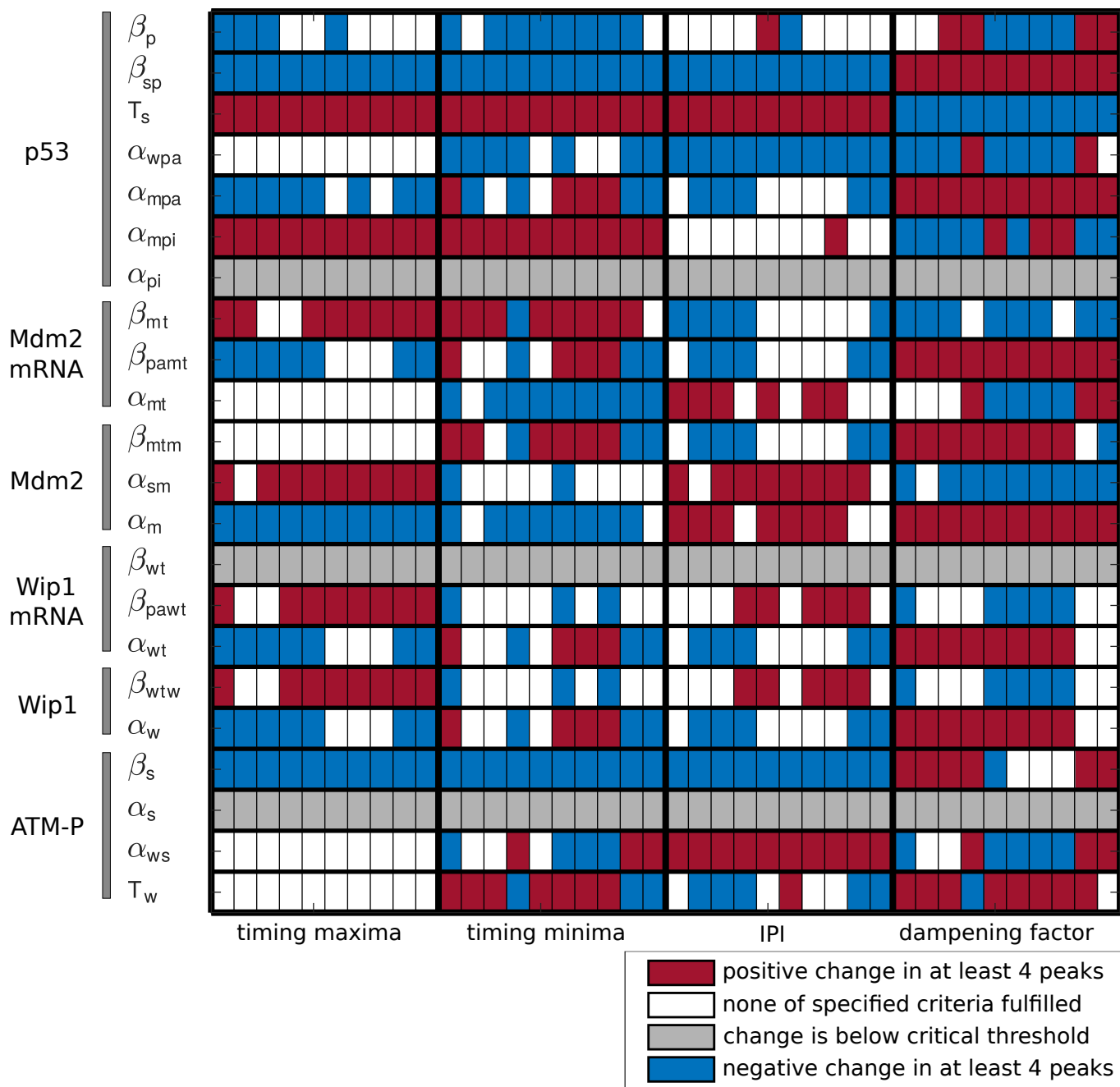

Supplement: S4 Fig — Changes in features that exceed a threshold of 1·10−3 are depicted in red if the change is positive and blue if the change is negative. Grey boxes indicate changes in features below 1·10−4. The corresponding parameter perturbation is considered to have no considerable effect. Sensitivity coefficients of peaks that are not consistently changed are depicted in white. (PDF) [file pcbi.1007901.s005.pdf]

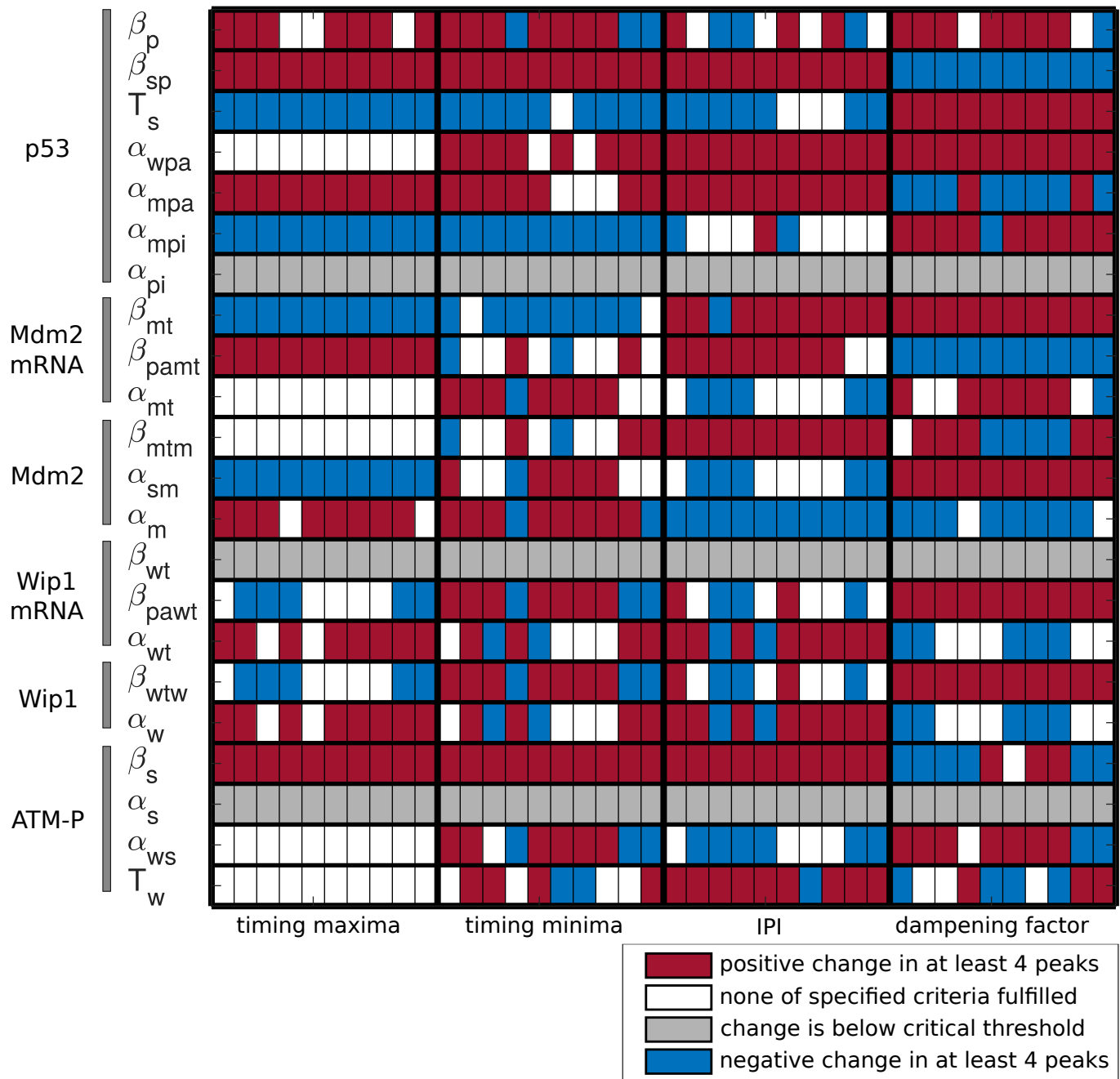

Supplement: S5 Fig — Changes in features that exceed a threshold of 1·10−3 are depicted in red if the change is positive and blue if the change is negative. Grey boxes indicate changes in features below 1·10−4. The corresponding parameter perturbation is considered to have no considerable effect. Sensitivity coefficients of peaks that are not consistently changed are depicted in white. (PDF) [file pcbi.1007901.s006.pdf]

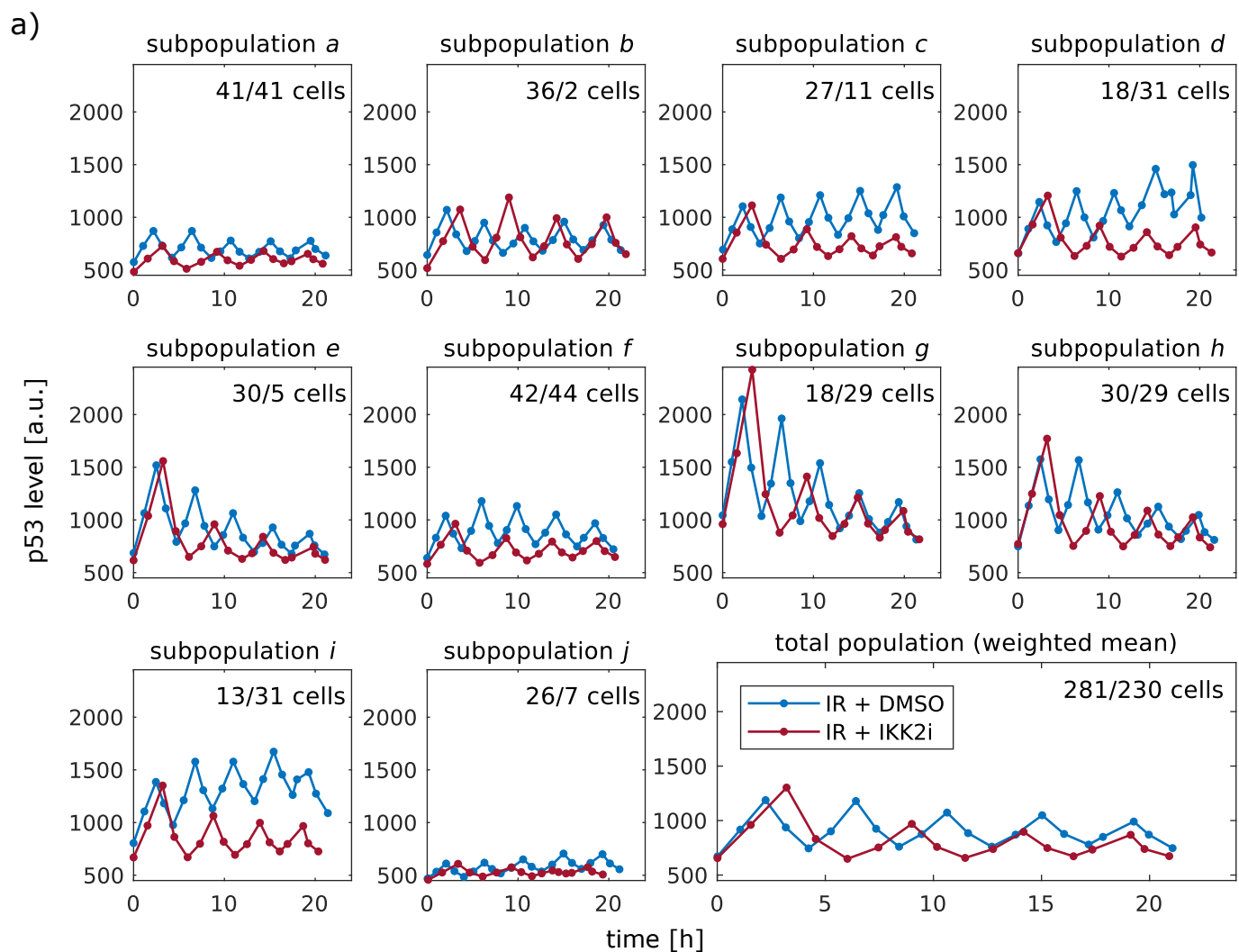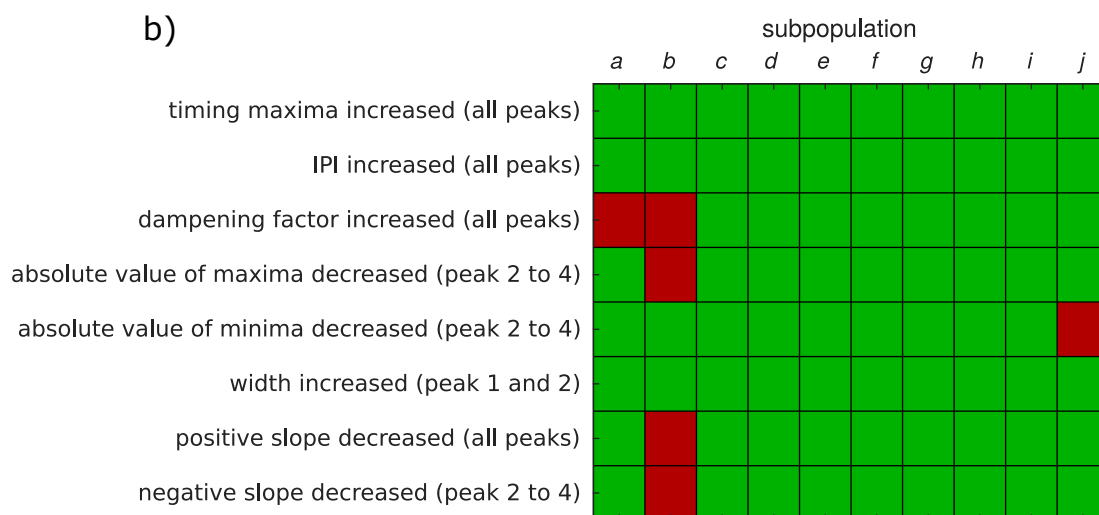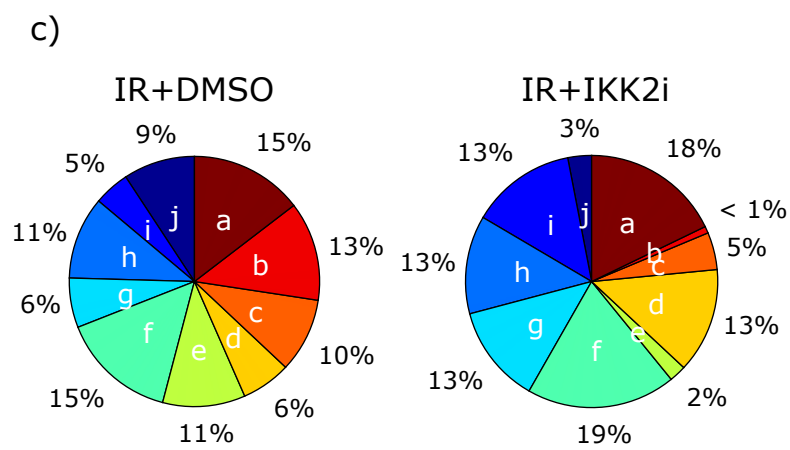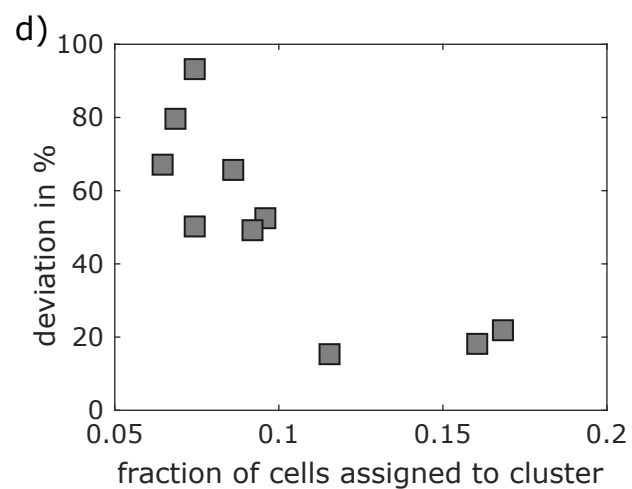

Supplement: S6 Fig — a) Blue dots and lines represent the peak-based mean of trajectories from the calibration data. The red dots and lines depict the peak-based mean of trajectories from the perturbation data, assigned to the respective subpopulation. The number of trajectories which are assigned to a subpopulation and used for computing the peak-based mean, are stated by the cell numbers in the upper right corner of each subpopulation plot. The first number denotes the subpopulation-specific number of cells for the calibration data, the second number accounts for the perturbation data. The peak-based mean for the total population is shown in the lower right corner of the figure and was determined by calculating the weighted mean over all subpopulations. The weight is given by the number of cells assigned to a subpopulation. b) Evaluation of fulfilled criteria of the peak-based mean of the perturbation data. Green boxes indicate that a certain criterion, described on the left-hand side, is fulfilled by an individual subpopulation (a to j). Red boxes represent unmet criteria. c) The percentages denote the relative amounts of cells assigned to a subpopulation for the calibration data (IR+DMSO) and the perturbation data (IR+IKK2i). d) The grey boxes represent the ten subpopulations. To determine the impact of a subpopulation on the model fit, the sum of cells assigned to a subpopulation of both conditions (IR+DMSO and IR+IKK2i) is normalized to the total amount of cells in both conditions. Deviation d for a subpopulation is determined by the difference in percentages (f) between both conditions which is normalized to the maximal percentage of the two conditions: d=|fIR-fIKK2i|max({fIR,fIKK2i})*100%. (PDF) [file pcbi.1007901.s007.pdf]

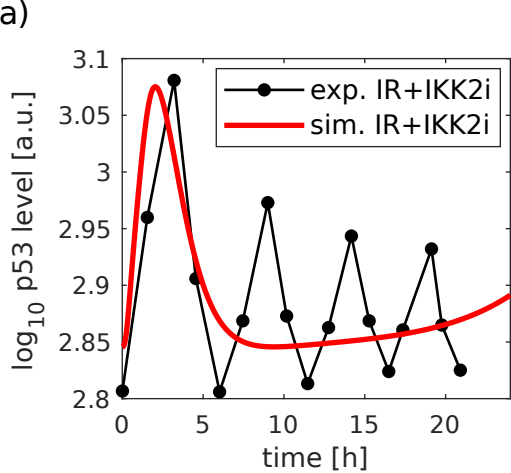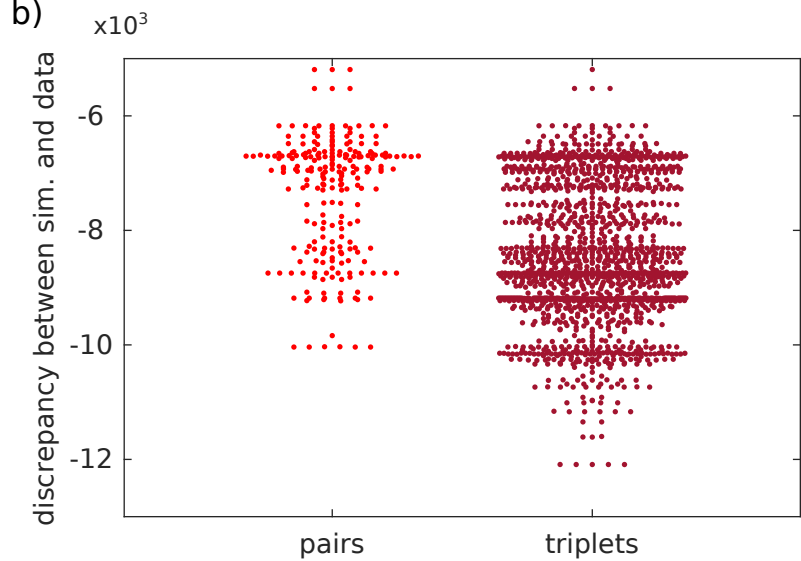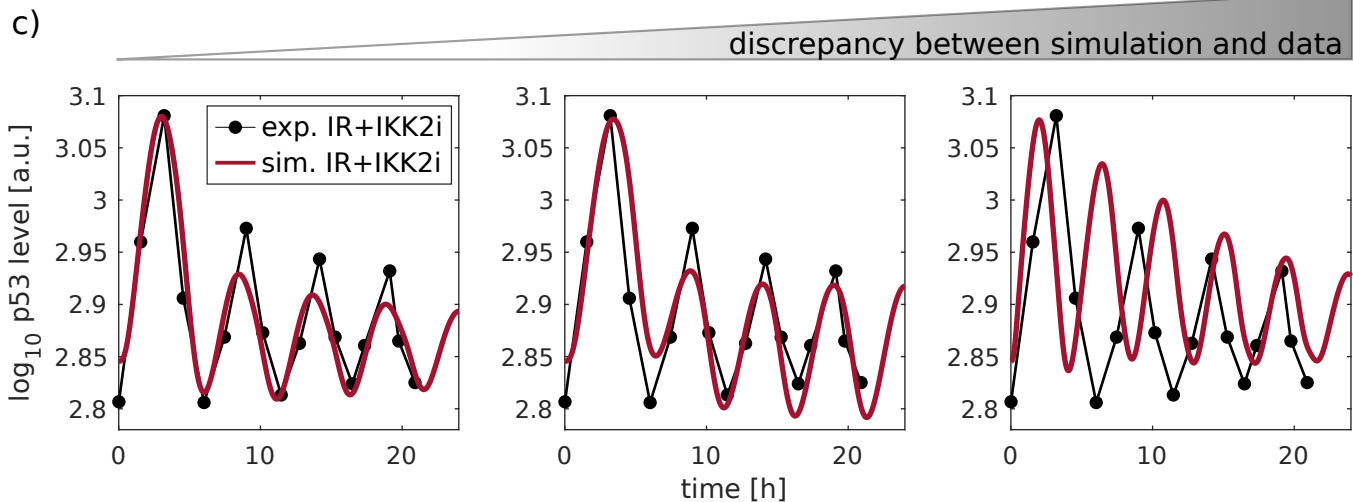

Supplement: S7 Fig — a) Simulation of the best fit of all tested parameter pairs. For a better visualization, the weighted mean over all subpopulations is shown for the simulation (red line) and the peak-based mean (black line with dots). b) Each dot represents a combination of parameter pairs (light red) or triplets (dark red) and the corresponding discrepancy between simulation and experimental data. c) The plots show simulations of three representative parameter combination fits, resulting in different fit qualities. (PDF) [file pcbi.1007901.s008.pdf]

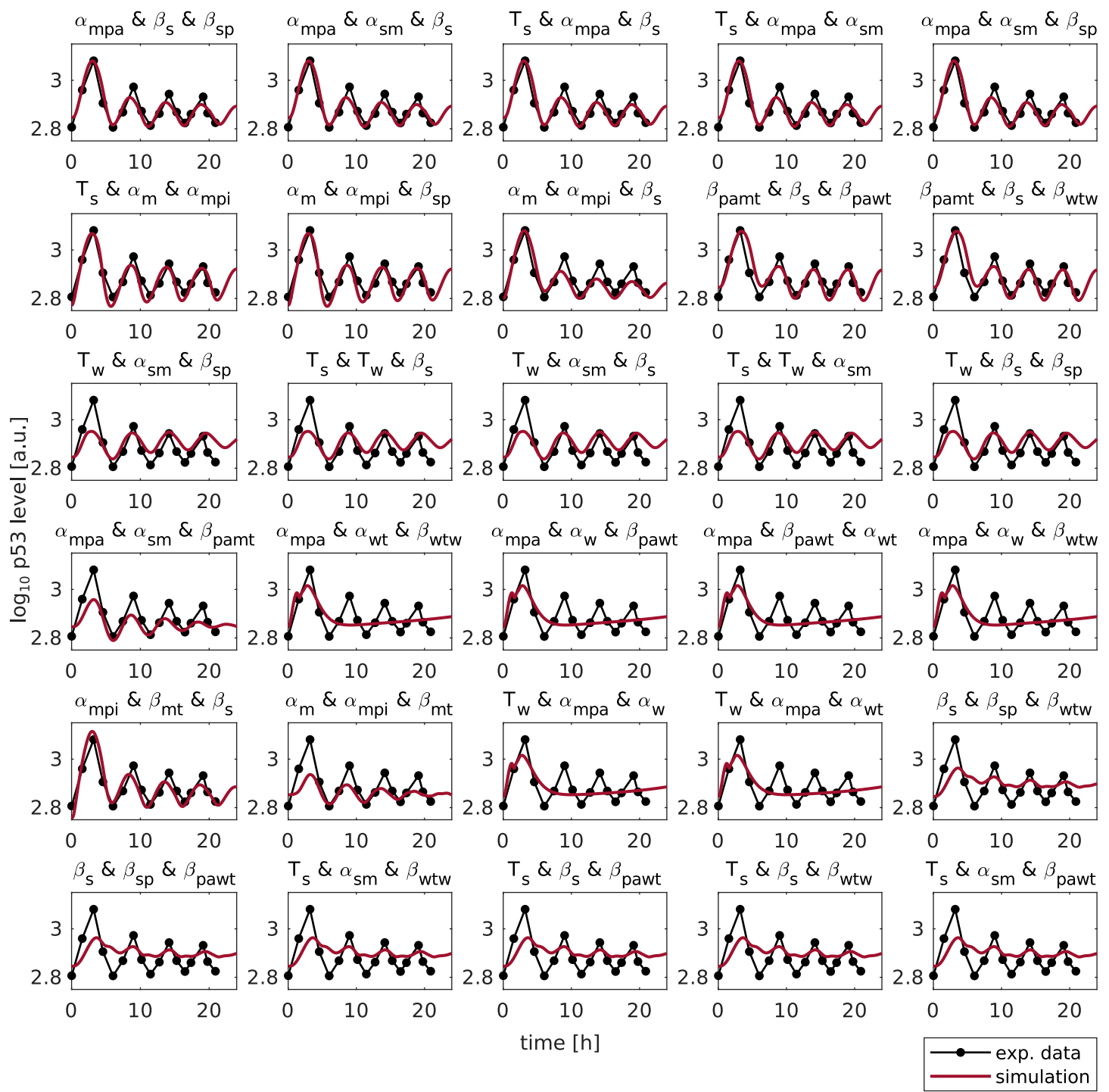

Supplement: S8 Fig — The black line with dots represents the peak-based mean. The red line depicts the simulation of the specified parameter combination fit. For a more compact visualization, the peak-based mean and the simulation of individual subpopulations is represented by the weighted mean, which is determined by averaging over all subpopulations. The weight is derived from the number of cells assigned to a subpopulation. (PDF) [file pcbi.1007901.s009.pdf]

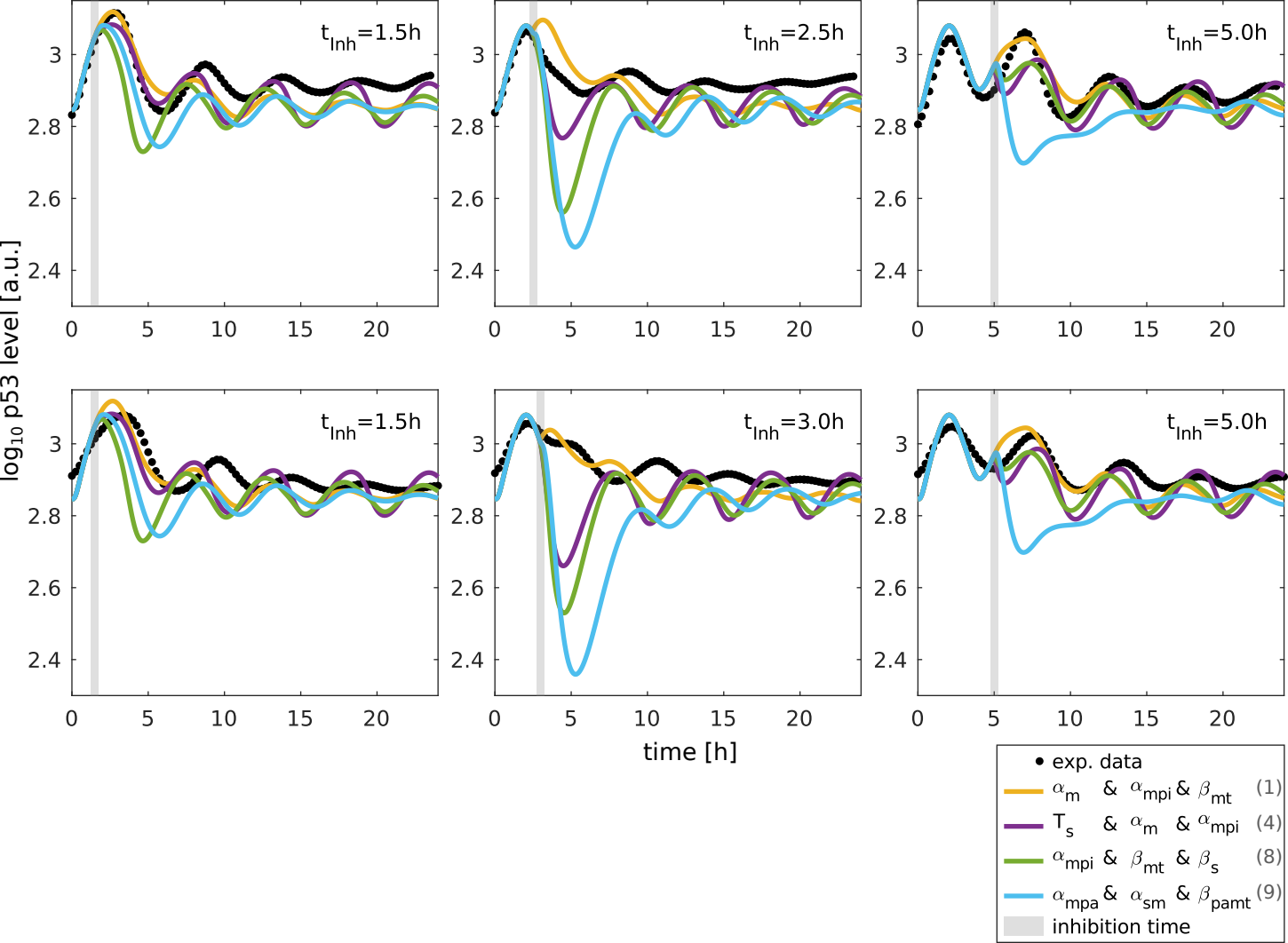

Supplement: S9 Fig — The experimental data (black dots) shows mean p53 dynamics upon IR and IKK2 inhibition at the specified time points. Simulations of four selected parameter combinations are represented by the colored lines, denoting the weighted mean of subpopulation dynamics. The index of each parameter combination derived from the corresponding summarized log10 χ2 value (Fig 5b) is given by the number in brackets. (PDF) [file pcbi.1007901.s010.pdf]
