# Supplementary material for: A systematic approach to decipher crosstalk in the p53 signaling pathway using single cell dynamics
Source: PLoS Comput Biol. 2020 Jun 26;16(6):e1007901. doi: 10.1371/journal.pcbi.1007901 (PMC7319280; doi:10.1371/journal.pcbi.1007901)
Supplement: S2 Fig — a) Western blot analysis of p53 and GAPDH upon 10 Gy IR in A549 cells treated with DMSO or IKK2i. b) Using live-cell time-lapse microscopy, A549 reporter cells were tracked and the p53 median nuclear fluorescence intensity was measured upon 0 Gy or 10 Gy IR in cells treated with DMSO or IKK2i. c) A549 reporter cells were tracked and the p53 median nuclear fluorescence intensity was measured upon 10 Gy IR in cells treated with DMSO or different IKK2 inhibitors. d) The specificity of IKK2 inhibition was tested by comparing the effect of the IKK2 inhibitor TPCA-1 on features of p53 dynamics with the effects of two structurally independent IKK2 inhibitors (BMS and SC). For comparison, 17 criteria were defined which are derived from altered features, induced by application of TPCA-1. A green box indicates that an inhibitor induced the same specified effect as the TPCA-1 inhibitor. A red box represents a mismatch for the observed effect of the specified inhibitor. e) Western blot analysis of pSTAT3 and GAPDH in A549 cells treated with DMSO or the corresponding IKK2i prior to IL-6 addition. f) Using live-cell time-lapse microscopy, A549 reporter cells were tracked and the p53 median nuclear fluorescence intensity was measured upon 10 Gy IR in cells treated with DMSO, IKK2i or IL-6. (PDF) [file pcbi.1007901.s003.pdf]

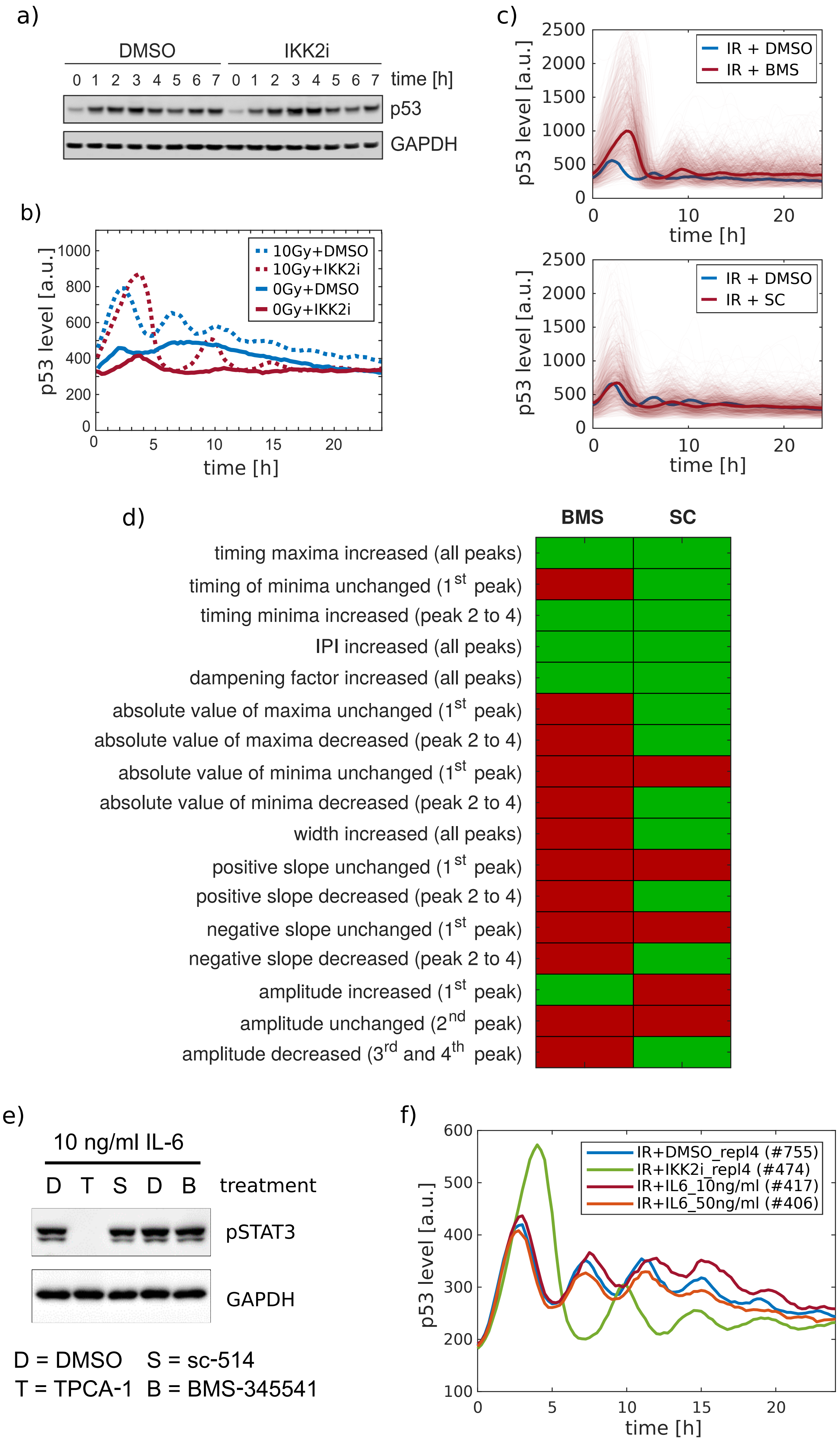
a)
DMSO
IKK2i
0 1 2 3 4 5 6 7 0 1 2 3 4 5 6 7 time [h]
p53
GAPDH
b)
10Gy+DMSO
10Gy+IKK2i
0Gy+DMSO
0Gy+IKK2i
p53 level [a.u.]
time [h]
c)
IR + DMSO
IR + BMS
IR + DMSO
IR + SC
p53 level [a.u.]
time [h]
d)
BMS
SC
timing maxima increased (all peaks)
timing of minima unchanged (1st peak)
timing minima increased (peak 2 to 4)
IPI increased (all peaks)
dampening factor increased (all peaks)
absolute value of maxima unchanged (1st peak)
absolute value of maxima decreased (peak 2 to 4)
absolute value of minima unchanged (1st peak)
absolute value of minima decreased (peak 2 to 4)
width increased (all peaks)
positive slope unchanged (1st peak)
positive slope decreased (peak 2 to 4)
negative slope unchanged (1st peak)
negative slope decreased (peak 2 to 4)
amplitude increased (1st peak)
amplitude unchanged (2nd peak)
amplitude decreased (3rd and 4th peak)
e)
10 ng/ml IL-6
D T S D B treatment
pSTAT3
GAPDH
D = DMSO S = sc-514
T = TPCA-1 B = BMS-345541
f)
IR+DMSO_repl4 (#755)
IR+IKK2i_repl4 (#474)
IR+IL6_10ng/ml (#417)
IR+IL6_50ng/ml (#406)
p53 level [a.u.]
time [h]
