# Supplementary material for: A systematic approach to decipher crosstalk in the p53 signaling pathway using single cell dynamics
Source: PLoS Comput Biol. 2020 Jun 26;16(6):e1007901. doi: 10.1371/journal.pcbi.1007901 (PMC7319280; doi:10.1371/journal.pcbi.1007901)
Supplement: S10 Fig — Western blot analysis of Wip1 and Mdm2 (a) as well as pChk2 (b) and GAPDH upon 10 Gy IR in A549 cells treated with DMSO or IKK2i. c) Summary of previously reported interactions between IKK2 and p53. (PDF) [file pcbi.1007901.s011.pdf]

a)

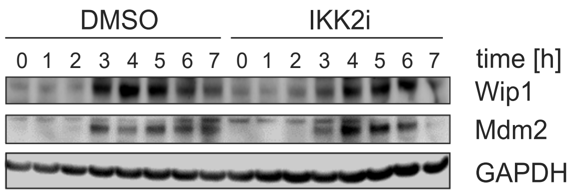

b)

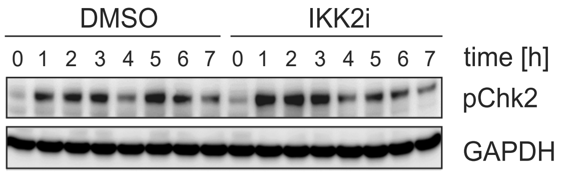

c) **Evidence of IKK2 specific crosstalk**

**Predicted effect of IKK2 inhibition**	**Reference**	**Description**
Reduced Mdm2-mediated degradation of active p53 (α_{mpa})	[32,33]	IKK2 promotes degradation of p53 independently of Mdm2
	[42,45-47]	IKK2 phosphorylates p53 at S392, which leads to ubiquitination by UBE4B and Mdm2-mediated degradation
Increased basal degradation of Mdm2 (α_m)	[48-50]	IKK2 phosphorylates Mdm2 at S166, which interferes with its translocation to the nucleus
Reduced ATM-dependent degradation of Mdm2 (α_{sm})	[34]	Suppressing NF-κB signaling by overexpressing a non-degradable IκBα mutant causes increased levels of Mdm2
Reduced ATM-mediated activation of p53 (β_{sp})	[42,51,52]	Blocking the IKK2-mediated phosphorylation of p53 at S392 delays the ATM-mediated phosphorylation of p53 at S15
