# Supplementary material for: A systematic approach to decipher crosstalk in the p53 signaling pathway using single cell dynamics
Source: PLoS Comput Biol. 2020 Jun 26;16(6):e1007901. doi: 10.1371/journal.pcbi.1007901 (PMC7319280; doi:10.1371/journal.pcbi.1007901)
Supplement: S1 Table — (PDF) [file pcbi.1007901.s012.pdf]

Description and estimated values of parameters of the calibrated model pool

label	$\log_{10}$ value		bounds	description
T_s	1.49	c	[-10.0, 4.0]	ATM-P-mediated activation of p53
T_w	1.12	c	[-10.0, 4.0]	Wip1-mediated dephosphorylation of ATM-P
α_m	$4 \cdot 10^{-14}$	h^{-1}	[0.0, 0.7]	basal degradation of Mdm2
α_{mpa}	-1.10	$c^{-1}\cdot h^{-1}$	[-10.0, 4.0]	Mdm2-mediated degradation of activated p53
α_{mpi}	-2.26	$c^{-1}\cdot h^{-1}$	[-10.0, 4.0]	Mdm2-mediated degradation of p53
α_{pi}	-10.00	h^{-1}	[-10.0, 4.0]	basal degradation of p53
α_s	-10.00	h^{-1}	[-10.0, 4.0]	basal dephosphorylation of ATM-P
α_{sm}	1.17	$c^{-1}\cdot h^{-1}$	[-10.0, 4.0]	ATM-P-induced degradation of Mdm2
α_w	$-4 \cdot 10^{-14}$	h^{-1}	[-1.0, 0.0]	basal degradation of Wip1
α_{wpa}	0.38	$c^{-1}\cdot h^{-1}$	[-10.0, 4.0]	Wip1-mediated dephosphorylation of active p53
α_{ws}	4.00	h^{-1}	[-10.0, 4.0]	Wip1-mediated dephosphorylation of ATM-P
β_{pamt}	1.98	h^{-1}	[-10.0, 4.0]	transcription of Mdm2 gene induced by active p53
α_{mt}	-0.28	h^{-1}	[-1.0, 0.0]	degradation of Mdm2 transcript
β_{mt}	1.33	$c\cdot h^{-1}$	[-10.0, 4.0]	basal transcription of Mdm2 gene
β_{mt}-fca	-0.01		[-1.5, 1.5]	
β_{mt}-fcb	-0.17		[-1.5, 1.5]	
β_{mt}-fcc	$2 \cdot 10^{-19}$		[-1.5, 1.5]	
β_{mt}-fcd	$7 \cdot 10^{-20}$		[-1.5, 1.5]	
β_{mt}-fce	-0.42		[-1.5, 1.5]	subpopulation-specific fold change
β_{mt}-fcf	0.22		[-1.5, 1.5]	
β_{mt}-fcg	$2 \cdot 10^{-19}$		[-1.5, 1.5]	
β_{mt}-fch	0.16		[-1.5, 1.5]	
β_{mt}-fci	0.11		[-1.5, 1.5]	
β_{mt}-fcj	-0.29		[-1.5, 1.5]	
β_{mtm}	0.37	h^{-1}	[-10.0, 4.0]	translation of Mdm2 transcript
β_{mtm}-fca	-0.19		[-1.5, 1.5]	
β_{mtm}-fcb	$2 \cdot 10^{-19}$		[-1.5, 1.5]	
β_{mtm}-fcc	$2 \cdot 10^{-19}$		[-1.5, 1.5]	
β_{mtm}-fcd	$7 \cdot 10^{-20}$		[-1.5, 1.5]	
β_{mtm}-fce	0.65		[-1.5, 1.5]	subpopulation-specific fold change
β_{mtm}-fcf	-0.19		[-1.5, 1.5]	
β_{mtm}-fcg	0.10		[-1.5, 1.5]	
β_{mtm}-fch	$7 \cdot 10^{-11}$		[-1.5, 1.5]	
β_{mtm}-fci	-0.10		[-1.5, 1.5]	
β_{mtm}-fcj	$7 \cdot 10^{-20}$		[-1.5, 1.5]	
β_p	0.36	$c\cdot h^{-1}$	[-10.0, 4.0]	synthesis of p53
β_p-fca	-0.26		[-1.5, 1.5]	
β_p-fcb	-0.16		[-1.5, 1.5]	
β_p-fcc	$-8 \cdot 10^{-03}$		[-1.5, 1.5]	
β_p-fcd	$2 \cdot 10^{-19}$		[-1.5, 1.5]	
β_p-fce	0.22		[-1.5, 1.5]	subpopulation-specific fold change
β_p-fcf	$-8 \cdot 10^{-12}$		[-1.5, 1.5]	
β_p-fcg	0.30		[-1.5, 1.5]	
β_p-fch	0.20		[-1.5, 1.5]	
β_p-fci	0.09		[-1.5, 1.5]	
β_p-fcj	-0.46		[-1.5, 1.5]	
β_s	0.28	$c\cdot h^{-1}$	[-10.0, 4.0]	DSB-induced activation of ATM

$\beta_s\text{-}fca$	0.05		[-1.5, 1.5]	subpopulation-specific fold change
$\beta_s\text{-}fcb$	0		[-1.5, 1.5]	
$\beta_s\text{-}fcc$	$-3 \cdot 10^{-3}$		[-1.5, 1.5]	
$\beta_s\text{-}fcd$	0.03		[-1.5, 1.5]	
$\beta_s\text{-}fce$	-0.24		[-1.5, 1.5]	
$\beta_s\text{-}fcf$	0.12		[-1.5, 1.5]	
$\beta_s\text{-}fcg$	-0.12		[-1.5, 1.5]	
$\beta_s\text{-}fch$	-0.12		[-1.5, 1.5]	
$\beta_s\text{-}fci$	0.03		[-1.5, 1.5]	
$\beta_s\text{-}fcj$	0.07		[-1.5, 1.5]	
β_{sp}	4.00	h^{-1}	[-10.0, 4.0]	ATM-P-mediated activation of p53
β_{pawt}	1.13	h^{-1}	[-10.0, 4.0]	transcription of Wip1 gene induced by active p53
α_{wt}	$-4 \cdot 10^{-14}$	h^{-1}	[-1.0, 0.0]	degradation of Wip1 transcript
β_{wt}	-10.00	$c \cdot h^{-1}$	[-10.0, 4.0]	basal transcription of Wip1 gene
$\beta_{wt}\text{-}fca$	$2 \cdot 10^{-19}$		[-1.5, 1.5]	subpopulation-specific fold change
$\beta_{wt}\text{-}fcb$	$2 \cdot 10^{-19}$		[-1.5, 1.5]	
$\beta_{wt}\text{-}fcc$	$2 \cdot 10^{-19}$		[-1.5, 1.5]	
$\beta_{wt}\text{-}fcd$	$2 \cdot 10^{-19}$		[-1.5, 1.5]	
$\beta_{wt}\text{-}fce$	$-8 \cdot 10^{-11}$		[-1.5, 1.5]	
$\beta_{wt}\text{-}fcf$	$2 \cdot 10^{-19}$		[-1.5, 1.5]	
$\beta_{wt}\text{-}fcg$	$2 \cdot 10^{-19}$		[-1.5, 1.5]	
$\beta_{wt}\text{-}fch$	$2 \cdot 10^{-19}$		[-1.5, 1.5]	
$\beta_{wt}\text{-}fci$	$2 \cdot 10^{-19}$		[-1.5, 1.5]	
$\beta_{wt}\text{-}fcj$	$2 \cdot 10^{-19}$		[-1.5, 1.5]	
β_{wtw}	-1.18	h^{-1}	[-10.0, 4.0]	translation of Wip1 transcript
$\beta_{wtw}\text{-}fca$	-0.09		[-1.5, 1.5]	subpopulation-specific fold change
$\beta_{wtw}\text{-}fcb$	0		[-1.5, 1.5]	
$\beta_{wtw}\text{-}fcc$	-0.05		[-1.5, 1.5]	
$\beta_{wtw}\text{-}fcd$	0.12		[-1.5, 1.5]	
$\beta_{wtw}\text{-}fce$	0.09		[-1.5, 1.5]	
$\beta_{wtw}\text{-}fcf$	-0.22		[-1.5, 1.5]	
$\beta_{wtw}\text{-}fcg$	-0.27		[-1.5, 1.5]	
$\beta_{wtw}\text{-}fch$	-0.26		[-1.5, 1.5]	
$\beta_{wtw}\text{-}fci$	0.08		[-1.5, 1.5]	
$\beta_{wtw}\text{-}fcj$	0.32		[-1.5, 1.5]	
n_s	$\log_{10}(4)$		[-, -]	ATM-P-mediated activation of p53
n_w	$\log_{10}(4)$		[-, -]	Wip1-mediated dephosphorylation of ATM-P
o_{ffset}	1.33		[-10.0, 3.0]	systematic shift (error model)
s_{cale_p53}	2.17		[-10.0, 4.0]	scaling factor (error model)
sd_{p53_a}	-1.82		[-3.0, 0.0]	subpopulation-specific standard deviation
sd_{p53_b}	-1.70		[-3.0, 0.0]	
sd_{p53_c}	-1.67		[-3.0, 0.0]	
sd_{p53_d}	-1.39		[-3.0, 0.0]	
sd_{p53_e}	-1.74		[-3.0, 0.0]	
sd_{p53_f}	-1.81		[-3.0, 0.0]	
sd_{p53_g}	-1.66		[-3.0, 0.0]	
sd_{p53_h}	-1.84		[-3.0, 0.0]	
sd_{p53_i}	-1.54		[-3.0, 0.0]	
sd_{p53_j}	-2.02		[-3.0, 0.0]	
